# The Impact of Genetic Factors in Ménière’s Disease

**DOI:** 10.3390/ijms27062788

**Published:** 2026-03-19

**Authors:** Iustin Mihai Iațentiuc, Otilia Elena Frăsinariu, Andreea Iațentiuc, Lucia Corina Dima-Cozma, Raluca Olariu, Luminița Mihaela Rădulescu, Ingrith Crenguța Miron, Iuliana Magdalena Stârcea, Cristina Gavrilovici, Cristina Mihaela Ghiciuc, Violeta Necula, Sebastian Romică Cozma, Oana Roxana Bitere-Popa

**Affiliations:** 1 Grigore T. Popa University of Medicine and Pharmacy, 700115 Iași, Romania; iatentiuc_iustin@yahoo.com (I.M.I.); andreea.iatentiuc@umfiasi.ro (A.I.); cozma.dima@umfiasi.ro (L.C.D.-C.); raluca.olariu@umfiasi.ro (R.O.); lmradulescu@yahoo.com (L.M.R.); ingridmiron@gmail.com (I.C.M.); iuliana.starcea@umfiasi.ro (I.M.S.); cri.gavrilovici@umfiasi.ro (C.G.); cristina.ghiciuc@umfiasi.ro (C.M.G.); sebastian.cozma@umfiasi.ro (S.R.C.); oana-roxana.bitere@umfiasi.ro (O.R.B.-P.); 2 Iuliu Hatieganu University of Medicine and Pharmacy Cluj-Napoca, 400012 Cluj-Napoca, Romania; violeta.necula@umfcluj.ro

**Keywords:** genetic factors, Ménière disease, genes, familial, sporadic

## Abstract

Ménière’s disease is an idiopathic disorder of the inner ear whose causes and pathogenetic mechanisms remain insufficiently elucidated. Advances in genetic analysis technologies have shifted attention toward the role of hereditary components in Ménière’s disease, generating a considerable number of studies investigating the relationship between human genetic variations and disease onset. The results of these investigations highlight the complex and heterogeneous nature of pathogenesis, involving both genetic and epigenetic alterations. Studies have identified multiple candidate genes involved in the regulation of endolymphatic fluid homeostasis, immune response, control of oxidative stress, and maintenance of the structural integrity of the inner ear. However, the reproducibility of these associations varies substantially across populations, reflecting the impact of ethnic heterogeneity, HLA haplotypes, and phenotypic diversity among patients. Moreover, non-genetic factors may act as triggers or modulators of the disease in genetically predisposed individuals. Taken together, the evidence supports a polygenic and context-dependent model of the disorder, in which genes influence susceptibility but do not directly determine disease development. Integrating genomic information with clinical data, analyzing immunological profiles, and investigating exposure to environmental factors are essential steps for patient classification and for the development of individualized treatment strategies.

## 1. Introduction

Ménière’s disease (MD) is a chronic and complex disorder of the inner ear, resulting from the interaction of multiple genetic and environmental factors. In 1861, Prosper Ménière presented a paper to the Academy of Medicine in Paris, marking an important milestone by identifying the inner ear as the source of the diverse symptoms that today define the disease bearing his name. There is growing evidence of a genetic predisposition, at least regarding familial cases [1,2].

**Figure 1:**
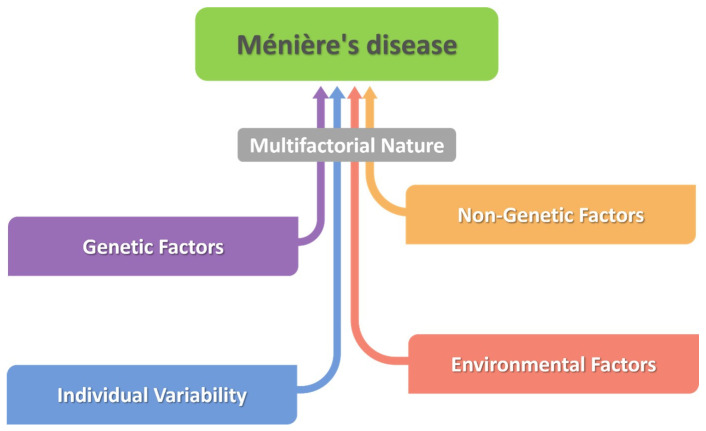
Multifactorial nature of Ménière’s disease.

Epidemiological and genetic studies indicate the presence of a hereditary predisposition, involving diverse biological pathways, including endocrine regulation, the innate immune response, and the autonomic nervous system. In addition, factors such as allergies, viral or bacterial infections, vascular disorders, or genetic mutations may disrupt inner ear fluid homeostasis, thereby contributing to the onset of symptoms. Currently, most specialists consider MD to be a multifactorial condition, resulting from the complex interaction between genetic and environmental factors (Figure 1) [2,3]. Despite extensive research, the pathophysiological mechanisms and determining factors of MD remain insufficiently understood. Part of this limitation is due to the difficulty of diagnosis, particularly in the early stages, when clinical manifestations are subtle and nonspecific [4,5].

Clinically, the disease manifests through recurrent episodes of vertigo, accompanied by sensorineural hearing loss (SNHL), tinnitus (ringing in the ears), and a sensation of aural fullness or pressure [2]. According to the diagnostic criteria established by the American Academy of Otolaryngology–Head and Neck Surgery (AAO-HNS, 1995), MD is defined by recurrent and well-demarcated episodes of rotational vertigo, accompanied by nystagmus, with a duration ranging from 20 min to 24 h. During these attacks, patients frequently experience nausea, vomiting, or both symptoms that reflect the severity of vestibular impairment. Sensorineural hearing loss has a fluctuating but progressive course, is most often unilateral, predominantly affects low or mid frequencies, and may be associated with aural fullness in the affected ear. In addition, tinnitus, usually unilateral, varies in intensity and tends to peak during vertiginous episodes, representing a characteristic feature of the clinical presentation. In some cases, headache may accompany vertigo attacks, and bilateral involvement is reported in approximately 25–40% of patients. For confirmation and follow-up, periodic audiological and vestibular investigations are recommended, as they are useful in assessing disease progression [2,5,6].

Classical genetic investigations in MD have focused on studying groups of individuals with a similar genetic background, in which some individuals were affected by the disease while others were not. The analysis of extended pedigrees allows the identification of the mode of inheritance and disease penetrance, while comparing the genomes of affected patients with those of healthy individuals facilitates the identification of genomic regions involved. The use of increasingly dense sets of genomic markers has improved analytical resolution, leading to the detection of potential candidate genes, and sequencing these genes can reveal mutations that alter gene function, thereby confirming their role in disease pathogenesis. This strategy is particularly effective for genes with major effects, in which a mutation in a single gene produces evident changes in the function of the systems involved [3,7].

In this context, the integrated analysis of genetic factors becomes extremely important for understanding the complex mechanisms that determine the onset and progression of MD, as well as for the development of personalized prevention and treatment strategies. Therefore, the evolution of diagnostic criteria and the accumulated clinical data underscore the complexity of MD and confirm that, in addition to environmental factors and pathophysiological mechanisms, genetic susceptibility plays an essential role in the development and progression of this disease [3,8].

This review article aims to synthesize and critically evaluate relevant studies published in recent years that highlight the role of genetic factors in MD. We analyzed candidate genes reported or investigated in scientific literature, with the objective of emphasizing both the potential benefits and the current limitations of genetic testing in MD. In the context of the clinical and genetic heterogeneity of the disease, numerous aspects require careful consideration by researchers and clinicians. Accordingly, we aim to clarify these elements and to provide a coherent framework for interpreting the available genetic data in MD.

## 2. Genetic Factors in Ménière’s Disease

For a long time, the genetic component of MD was overlooked, although many patients reported a family history of vertigo and hearing loss. Research has converged on the idea that MD has an important genetic component, supported by several independent lines of evidence [8,9]. The molecular mechanisms that regulate ionic homeostasis and fluid balance in the inner ear are essential for maintaining endolymphatic pressure. In this context, several genes involved in ion and water transport have been investigated as possible genetic causes of endolymphatic hydrops (EH), a central feature of MD [10,11,12,13]. Numerous candidate gene–focused studies have also suggested that variants in genes such as MICA, TLR10, and NFKB1 may influence the progression of hearing loss in patients with MD, indicating a possible involvement of immune and inflammatory pathways in pathogenesis [14,15,16,17].

**Figure 2:**
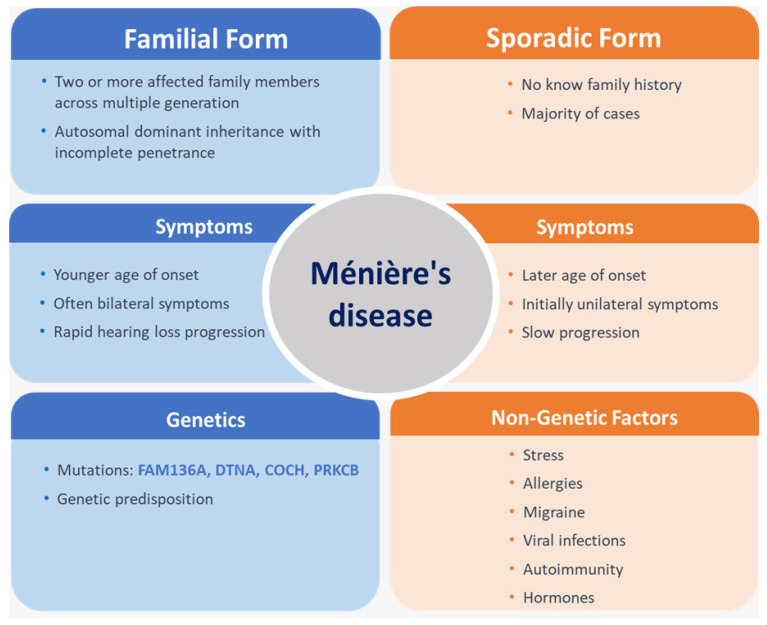
Familial Ménière’s Disease versus Sporadic Ménière’s Disease [8,18,19,20,21]. FAM136—Family with sequence similarity 136, member A; DTNA—Dytrobrevin alpha; COCH—Cochlin; PRKCB—Protein Ki-nase C Beta.

MD can be classified, from a clinical and epidemiological perspective, into familial forms (FMD) and sporadic forms (SMD) (Figure 2). The familial form is characterized by the presence of two or more affected members within the same family, often across multiple generations, suggesting a predominantly autosomal dominant (AD) inheritance pattern with incomplete penetrance. This form tends to have an earlier age of onset, morefrequently presents with bilateral symptoms, and may exhibit greater severity or a more rapid progression of hearing loss compared with SMD [18,19].

In contrast, the sporadic form accounts for most MD cases and occurs in individuals without known family history. Symptom onset is generally later, initial involvement is often unilateral, and progression to bilateral involvement is slower. Non-genetic factors appear to play a more prominent role in these cases. Thus, the main difference between FMD and SMD lies in the influence of genetic factors on disease susceptibility and severity, as well as in the way environmental factors interact with individual predisposition [20,21]. In the study conducted by Lee et al., the main clinical differences between SMD and FMD were age at onset and a higher frequency of migraine, findings that were similar in both Caucasian and Asian populations. Patients with definite FMD exhibited a significantly earlier disease onset compared with those with SMD [22].

From a molecular perspective, MD is considered a complex, multifactorial, and polygenic disorder in which variations in genes are involved in the maintenance of endolymphatic homeostasis, ion channel function, epithelial barrier integrity, regulation of the immune response, and control of oxidative stress may act cumulatively, promoting the development of EH and the characteristic clinical manifestations. The marked phenotypic variability observed among patients, even within the same family, suggests that genetic factors do not act in isolation but interact with one another and with environmental factors, thereby modulating the threshold for disease onset and severity. However, the results obtained are often inconsistent across populations, reflecting the genetic heterogeneity of the disease and underscoring the need for integrated genomic approaches involving large sample sizes and rigorous clinical characterization [3,9,20].

The genetic basis of MD can vary in some isolated families, rare forms of monogenic inheritance occur, but in many cases (familial or sporadic), a polygenic inheritance is assumed. The presence of familial forms has been consistently reported over the decades. Several studies have shown a link between a family history of hearing loss or episodes of vertigo and the occurrence of the disease [13,18,19,22]. For example, Requena et al. identified a positive family history in 34% of patients in a large study conducted in Spain and Italy (cohort of 1.245 patients), and familial cases accounted for approximately 8% of cases initially considered sporadic [19]. The risk of recurrence supports this predisposition. MD occurs 16–48 times more frequently in siblings of patients and 4–12 times more frequently in descendants compared with the general population [8]. This familial aggregation suggests an important role for genetic factors. Older studies have also reported a positive family history [23,24,25]. These studies provided a solid foundation and reinforced the hypothesis of a genetic cause of the disease, alongside environmental factors.

The unequal distribution among different ethnic groups (with a higher prevalence among Caucasian populations) further supports the existence of predisposing genetic factors [26]. Thus, current evidence outlines a coherent picture in which MD arises on the background of a complex genetic susceptibility, modulated by environmental factors and multisystem biological mechanisms.

### 2.1. Sporadic Ménière’s Disease

Sporadic Ménière’s disease refers to cases in which patients have no known relatives affected by the condition. In SMD, the classic symptoms appear in isolation, without an evident family history. However, in some families, patients’ relatives may exhibit only partial manifestations, such as hearing loss or isolated episodes of vertigo, indicating that the MD phenotype can be variable and fragmented, even within the same family. This variability makes it more difficult to identify a direct genetic link [21].

Genetic testing in SMD has recently focused on identifying rare variants using targeted exome sequencing techniques. Although the disease does not show obvious familial transmission, rare mutations or combinations of mutations in multiple genes may contribute to individual susceptibility, potentially influenced by recessive or de novo mutations. To detect these variants, population-specific datasets must be used, considering the genetic background of patients and controls, as the frequency of rare variants can vary significantly between populations. Ignoring this population stratification may lead to false-positive results [8,27]. Thus, SMD can be considered, at least in part, a polygenic disorder in which multiple genetic variants collectively contribute to the manifestation of symptoms, some of which are also present in familial forms. In addition, environmental and epigenetic factors may modulate the expression of these genes, explaining the isolated onset of the disease in individuals with no apparent family history [28].

In SMD, the genetic landscape is complex and involves multiple rare variants in genes associated with sensorineural hearing loss (GJB2, USH1G, SLC26A4, ESRRB, CLDN14) and in genes involved in axonal guidance signaling pathways (NTN4, NOX3). Gallego-Martínez et al. identified an accumulation of rare variants in hearing-loss–associated genes in Spanish patients with MD compared with frequencies in the Spanish and non-Finnish European populations [21]. These genes include those involved in the ionic regulation of endolymph, such as SLC26A4 and CLDN14, as well as genes associated with autosomal dominant or recessive monogenic deafness, such as GJB2 and ESRRB, and genes implicated in syndromic hearing loss, for example, USH1G. The excess of rare variants in these genes may influence the phenotypic expression of hearing loss in SMD and suggests interactions between different proteins, accounting for the clinical variability observed [8,21].

Moreover, rare variants have also been found in genes involved in axonal guidance signaling, such as NTN4 and NOX3, suggesting the involvement of trophic pathways and sensory cell innervation in SMD. It remains unclear whether these excess rare variants are specific to the Spanish population or are also present in other groups, as some hearing-loss–associated genes contain population-specific variants, which can affect their interpretation [21,29]. Additionally, in Asian and European cohorts, some of these variants have been reclassified as variants of uncertain significance (VUS) based on allele frequency differences across populations. In conclusion, Spanish patients with MD appear to carry a higher burden of VUS and pathogenic variants in genes such as ESRRB, SLC26A4, USH1G, CLDN14, and GJB2 compared with the reference Spanish population, based on the study by Gallego-Martinez [21,29].

Oh et al. identified 15 rare heterozygous variants in candidate genes previously associated with MD in 12 sporadic patients [30]. Studies using targeted sequencing panels have reported a burden of missense variants in several genes, including NOX3 and NTN4, as well as OTOG (which encodes components of the inner ear extracellular matrix). Other implicated genes, such as OTOGL, OTOP1, TECTA, ZNF91, and ARMC9, have been associated with vertigo in a recent genome-wide meta-analysis [31].

SMD and FMD appear to represent two manifestations of the same etiologic continuum, differentiated not by the nature of the mechanisms involved but by their relative weight. Familial forms clearly demonstrate the presence of a consolidated genetic inheritance, in which rare high-impact mutations or specific combinations of variants can be transmitted across generations, leading to earlier onset and a more severe phenotype. In contrast, sporadic presentations, although lacking an apparent family history, suggest the contribution of a complex genetic architecture, composed of rare variants with small to moderate effects, subtle interactions, and cumulative influences of environmental factors. These can trigger the disease even in the absence of an easily recognizable inheritance pattern. Thus, SMD does not necessarily represent a distinct entity but may reflect a combination of partially penetrant genetic predispositions, modulated by external exposures and individual factors [21,32].

This unified perspective, also supported by the observations of Galego et al. emphasizes that the strict distinction between FMD and SMD may be artificial [8]. To accurately decipher the genes involved, molecular networks, and pathophysiological mechanisms, future genetic studies must consider both the high phenotypic variability of the disease and the population structure, including multigenerational families, as well as the use of well-defined sporadic cohorts. Only by integrating these dimensions (genetic, clinical, and environmental) will it be possible to construct a coherent understanding of the etiology and progression of MD.

### 2.2. Monogenic Familial Ménière’s Disease

Monogenic FMD refers to a form of the disorder in which the condition is predominantly determined by a single gene, whose pathogenic variant is sufficient to cause the disease, independently or almost independently of other genetic or environmental factors. In this context, “monogenic” means that a mutation in a specific gene has a major effect, directly impacting inner ear function. These forms are identified almost exclusively in families with clear disease aggregation, where multiple affected members exhibit a consistent pattern of inheritance [18,33].

Genetic analysis of FMD has focused primarily on identifying monogenic causes using exome sequencing. In this context, rare genetic variants have been investigated, characterized by very low frequency in the general population, as well as by a frequency lower than the estimated prevalence of MD in the European population [8]. The results suggest that MD is a genetically heterogeneous disease, meaning that different mutations in different genes can cause the disorder in different families. Furthermore, patients within the same family may present with variable symptoms, such as age of onset and severity, a phenomenon known as variable expressivity [19].

Genetic heterogeneity is a well-recognized phenomenon in numerous human disorders, where similar clinical phenotypes can arise from mutations in distinct genes or even from phenocopies. A classic example comes from familial breast cancer, in which mutations in BRCA1, located at 17q21, and BRCA2, located at 13q12–q13, can produce nearly identical clinical manifestations, even though these genes are not allelic. By analogy with other complex diseases, it is highly likely that a similar degree of genetic heterogeneity characterizes MD as well [1]. Most families with MD follow an AD inheritance pattern with incomplete penetrance. This suggests that, in addition to genes, other modifying factors (whether genetic or environmental) also influence how the disease manifests. Although the AD pattern is the most frequently observed, mitochondrial or recessive transmission mechanisms have also been described, highlighting the etiologic complexity of the disease [18,19,33].

Another relevant genetic aspect of FMD is anticipation, a phenomenon in which the disease manifests at progressively younger ages in successive generations. At the molecular level, this process is attributed to DNA sequence instability, particularly trinucleotide repeats such as CAG triplets. As the number of these repeats increases beyond a critical threshold, disease susceptibility is amplified, and both severity and age of onset correlate with the length of the expansion. Unstable repeats can be in both exons and adjacent intronic regions, and the mechanism is well documented in disorders such as Huntington’s disease, myotonic dystrophy, and fragile X syndrome [33]. Interestingly, familial forms of MD have shown features compatible with this type of genetic mechanism, suggesting that anticipation may contribute to the observed clinical variability. When onset occurs in childhood or adolescence, clinical presentations tend to be bilateral and more severe. Within the same family, differences in age of onset are relatively small, rarely exceeding 6–7 years, and this phenomenon has also been confirmed by the observations of Arweiler et al. [33,34].

The first observations related to FMD were reported by Brown, who described it in two siblings from the same family, and subsequent studies provided additional evidence for the involvement of genetics in this disease [22]. A Finnish study reported that the prevalence of definite familial forms of MD was 9.3%, increasing to 23.4% when probable FMD cases were also included. In these cases, inheritance was largely AD with incomplete penetrance, identified in 87.5% of definite cases [35]. In the United Kingdom, approximately 5% of MD cases were considered familial, with an inheritance pattern still predominantly AD and more frequent mother-to-daughter transmission [33]. In Germany, the incidence of FMD was reported at 19.2%, and these families showed a high frequency of migraines (48.1%) [18]. In Spain, the frequency of FMD was 8%, with AD inheritance and anticipation; the only clinical difference between FMD and SMD was an earlier onset of the disease in familial forms [19].

In the study by Lee et al. in a Korean population, the inheritance pattern was predominantly AD with incomplete penetrance, with only 2 families out of 28 studied showing AR inheritance. The causes of incomplete penetrance remain unclear, with several hypotheses proposed (somatic mutations, multiple mutations, or involvement of mitochondrial DNA), but none have been confirmed. The study also highlighted a predominance of female cases and frequent mother-to-daughter transmission, suggesting the possibility of mitochondrial inheritance. However, this is difficult to demonstrate due to the lack of complete genealogical data (because of the death of previous generations and missing medical records) [22].

The disease can be transmitted through both maternal and paternal lines, making a strictly mitochondrial mechanism unlikely, except for the possibility of heteroplasmic variations. Nevertheless, correlating with the higher proportion of affected women, FMD appears to be transmitted approximately twice as frequently from mother to child as from father to child [1,33]. An interesting observation, still without a complete explanation, is the apparently uneven distribution of transmission: the disease is predominantly passed from mother to daughter, while boys tend to inherit the condition more often from fathers [36]. Although this pattern could suggest the involvement of a genomic imprinting mechanism, this is unlikely, as transmission has been documented from either parent [1,36].

Several chromosomal regions and genetic variants have been suggested as potentially involved in the onset of FMD; however, the exact genes and the pathological mechanisms determined by these variants require confirmation despite existing studies [22]. Major progress was achieved through exome sequencing, when Requena et al. identified the first two pathogenic mutations in a family from southeastern Spain, located in the FAM136A and DTNA genes [37]. Mutations in these genes perfectly co-segregated with all disease cases in the family. DTNA encodes α-dystrobrevin, a protein involved in the function of the blood–brain barrier, while FAM136A encodes a mitochondrial protein expressed in the cochlea and utricle during fetal development, possibly essential for normal inner ear formation [37]. These mutations are transmitted in an AD manner, persist across multiple generations, and exhibit genetic anticipation, reinforcing their involvement in familial forms of the disease. However, these findings have not yet been replicated in other families with MD.

Martin-Sierra et al. identified a candidate variant in the PRKCB gene in another Spanish family with MD, co-segregating with the low-frequency hearing loss phenotype. This gene encodes a protein kinase C beta, expressed in the mouse cochlea, with important roles in regulating cell growth, apoptosis, and immune response. The protein is expressed in the tectorial cells and inner margin cells [38]. Two other missense variants were identified in the SEMA3D and DPT genes in two different Spanish families, illustrating the genetic heterogeneity of familial cases. SEMA3D encodes a semaphorin involved in axonal guidance signaling and neuronal growth, while DPT is important for gap junction formation and the extracellular matrix [39].

Skarp et al. observed in a Finnish family, in a patient with early-onset MD, two rare mutations shared between grandfather and grandson, in the HMX2 gene, not previously reported, and TMEM55B, which has a very low frequency in the Finnish population. These shared variants support the role of rare variants in early-onset MD. However, the grandson also carried eleven additional mutations that were not present in the grandfather, suggesting a multiallelic contribution (multiple mutations acting together) and the possibility of genetic anticipation, with the disease worsening in younger generations [40].

Another study by Skarp et al. investigated the genetic basis of MD through a detailed analysis of three affected Finnish families, aiming to identify rare variants with potential pathogenic roles. In this analysis, seven rare genetic variants were detected, four of which were integrated into a co-expression network that includes genes previously associated with the disease. This observation suggests the existence of common functional modules involved in biological processes essential for maintaining inner ear homeostasis and normal vestibulocochlear function [32].

In the first family analyzed, the authors identified the variant c.200C>T/p.T67M (rs138264188) in the CYP2B6 gene, a member of the cytochrome P450 superfamily. Cytochromes P450 play a central role in cellular metabolism, being involved in the biotransformation of eicosanoids, mitochondrial respiration, and redox reactions necessary for the detoxification of endogenous and exogenous compounds. These enzymes also contribute to neutralizing reactive oxygen species (ROS), limiting oxidative stress at the cellular level [32,41]. Impaired cytochrome P450 function has previously been associated with various congenital anomalies, including auditory disorders and hearing loss [42]. Furthermore, polymorphisms in genes involved in the oxidative stress response, such as CYP1A1, have been correlated with presbycusis, suggesting an important role for these metabolic pathways in auditory degeneration. At the molecular level, excessive ROS accumulation can induce mitochondrial dysfunction and activate apoptotic pathways in cochlear hair cells, mechanisms described in both age-related hearing loss and auditory damage induced by noise or ototoxic agents [32,43].

From a pathophysiological perspective, increased oxidative stress and inflammatory activation may impair the function of the endolymphatic sac epithelium, a structure essential for endolymph resorption and the maintenance of fluid balance within the membranous labyrinth. Oxidative injury at this level can compromise ionic transport systems and epithelial integrity, thereby promoting the persistence of endolymphatic imbalance and the development of hydrops. In addition, endothelial dysfunction induced by oxidative stress may disrupt cochleo-vestibular microcirculation, further contributing to impaired tissue oxygenation and metabolic instability of the inner ear [32,42]. Another potential mechanism linking CYP2B6 to MD relates to its role in drug metabolism. Variability in CYP2B6 activity may alter the pharmacokinetics of medications commonly used in patients with vestibular disorders, migraine, or psychiatric comorbidities. Such variability may influence therapeutic response, adverse-effect profiles, and neurovascular regulation, thereby indirectly modulating symptom expression and disease progression [32,42,43].

A particularly relevant aspect for MD is that the expression and activity of cytochromes P450 can be modulated by external factors such as diet, chemical exposure, medications, or other environmental influences. This interaction between genetic background and environmental factors provides a plausible explanation for the fluctuating and episodic nature of symptoms observed within the same family. In this context, CYP2B6 emerges as a candidate gene of major interest, with potential functional relevance for genetic susceptibility and clinical variability in MD [32,41].

In the second family studied, a different molecular architecture was observed, with two distinct variants: c.323C>T/p.P108L in GUSB and c.1089G>A/p.W363X in EPB42. GUSB encodes β-glucuronidase, a lysosomal hydrolase involved in the degradation of glycosaminoglycans, which are key components of the extracellular matrix. Loss-of-function mutations in this gene cause mucopolysaccharidosis type VII, a lysosomal storage disorder characterized by skeletal, cognitive, and cardiac abnormalities, as well as hearing loss [44]. Experimental mouse models have suggested that sensorineural hearing loss results from changes in the mass and stiffness of cochlear structures, as well as impaired function of sensory cells, indicating possible vestibular involvement [45]. In contrast, EPB42, which is predominantly expressed in blood and involved in maintaining erythrocyte shape, does not appear to have a functional role in the inner ear and is not integrated into relevant functional interaction networks, making it unlikely to be a causative genetic factor for FMD. This distinction between genes highlights the importance of functional contextualization of genetic variants when evaluating them as potential determinants of disease [32].

The study of the third family provides an example of even greater genetic complexity, with four rare variants identified in four distinct genes, each with functional implications for cellular mechanisms critical to auditory and vestibular function. SLC6A7, a solute carrier, transports essential molecules such as amino acids, vitamins, and ions, and is part of a large family of membrane proteins involved in cellular homeostasis. Other members of this family, such as SLC26A4 and SLC26A5, are already associated with non-syndromic hearing loss, and imbalances in the activity of these transporters can induce oxidative stress and inflammation, as demonstrated in experimental models [46,47,48]. This makes SLC6A7 a promising genetic candidate for MD susceptibility [32]. Two heterozygous variants were also identified in genes involved in mitotic spindle organization: ASPM (c.5207A>G/p.Q1736R) and KNTC1 (c.5242A>C/p.T1748P). ASPM is known for its role in primary microcephaly and may be associated with hereditary hearing loss [49], while KNTC1 is essential for the assembly of the dynein-dynactin complex on kinetochores, crucial for ciliary motility and otolith biogenesis in the inner ear [50]. In contrast, OVCH1, although studied, shows no functional evidence related to hearing or balance and is not integrated into relevant molecular networks, suggesting that its involvement in the disease is unlikely [32].

To date, these variants appear to be unique to the families studied. The analysis of these rare genetic variants emphasizes that FMD cannot be explained by a single gene or mechanism. Rather, it results from a complex interaction between genetic factors and multiple biochemical mechanisms. This complexity accounts for the phenotypic variability observed between families and among members of the same family and suggests that susceptibility to MD involves both genetic predisposition and environmental factors [32]. Genetic studies have advanced significantly; however, no single gene has been identified as responsible for the entire pathology.

Phenotypic variability complicates genetic analysis and makes it difficult to exclude or validate candidate genes based on the genetic status of apparently healthy relatives. Furthermore, the onset of the disease in middle age creates challenges in accurately establishing the clinical status of younger family members, which can influence the interpretation of gene segregation [32]. To confirm and generalize these findings, replication studies in independent datasets or in other affected families would be ideal. Additionally, to understand the biological impact of these variants on disease pathogenesis, complementary experiments (both in vitro and in vivo) are required to clarify how these genes influence cellular function and the integrity of cochlear and vestibular structures. Table 1 illustrates the genes associated with FMD with AD inheritance.

### 2.3. Multiallelic Inheritance in Familial Ménière’s Disease

Multiallelic inheritance in FMD represents a rapidly expanding field, marked by considerable genetic complexity. Current data suggest that the clinical phenotype of familial forms cannot be explained by a single major gene, but rather by the simultaneous contribution of multiple rare variants that interact with each other and with environmental factors. In this sense, FMD appears to reflect a multiallelic inheritance model, where moderate-effect mutations distributed across different genes involved in inner ear homeostasis may cumulatively lead to disease development [8].

Although exome sequencing in multiplex families remains limited (currently fewer than 100 familial cases have been genetically investigated), recent research has begun to reveal recurring patterns [8]. A notable example is the identification of an increased burden of rare variants in the OTOG gene in a cohort of 15 unrelated Spanish families [55]. The OTOG gene, which encodes otogelin (a structural glycoprotein essential for the integrity of the tectorial membrane and the otolithic matrix) plays an important role in mechanical transduction and vestibulocochlear stability. Therefore, its disruption by rare variants may affect both auditory and vestibular function [8].

Although complete segregation of these variants could not be demonstrated for all families studied, primarily due to the inability to obtain DNA samples from all affected members, a remarkable aspect is the repeatability of these mutations: most rare variants were independently identified in 2–4 individuals from different multiplex families. This observation reinforces the hypothesis that OTOG plays an important role in genetic predisposition to FMD, rather than being a random result of genetic variability [8,55].

The clinical phenotype of patients carrying rare OTOG variants is characterized by rapid and severe auditory progression. According to published data, bilateral sensorineural hearing loss often begins around 60 dB within the first year of disease onset, gradually affecting the entire frequency spectrum. In many cases, hearing deterioration is uniform, reflecting a diffuse pattern of cochlear involvement, which suggests a profound disruption of intracochlear mechanisms associated with otogelin-dependent structures [8]. These findings support the notion that in FMD, inheritance does not necessarily follow a simple Mendelian pattern, but involves a heterogeneous genetic architecture, where multiple variants with small or moderate effects act cumulatively. Furthermore, affected individuals may carry unique combinations of mutations, explaining the phenotypic variability observed between families and even among members of the same family. The role of these rare variants must, however, be interpreted in the context of possible gene–gene and gene–environment interactions, which may amplify or attenuate phenotypic expressivity [8].

In the long term, understanding this multiallelic model has important implications for the diagnosis and management of FMD. The identification of recurrent rare variants, such as those in OTOG, paves the way for the development of dedicated genetic panels, which could facilitate early diagnosis in families with a history of MD, as well as patient stratification based on their genetic profile and risk of disease progression. Additionally, these findings support the concept that FMD results from the cumulative effect of subtle molecular dysregulations, rather than from a single major-effect mutation.

### 2.4. The COCH Gene, DFNA9, and Ménière’s Disease

Another genetically interesting example is autosomal dominant non-syndromic hearing loss type 9 (DFNA9), a hereditary form of adult-onset sensorineural hearing loss, often associated with vestibular symptoms similar to those seen in MD. DFNA9 is located on chromosome 14q12-13 and is caused by mutations in the COCH gene, which encodes cochlin, the most abundant protein in the human and mouse cochlea. Cochlin is essential for the structural integrity of the extracellular matrix and for the proper functioning of the auditory and vestibular systems. Rare COCH gene mutations have been identified in FMD and are associated with progressive hearing loss and recurrent episodes of vertigo, supporting its role as a susceptibility gene with a direct impact on disease pathophysiology. The phenotypic variability observed even within the same family suggests the influence of other genetic factors and environmental modifiers on clinical expression [9,56,57].

The characteristic auditory phenotype of DFNA9 manifests as early-onset high-frequency sensorineural hearing loss, accompanied by variable vestibular dysfunction, ranging from recurrent vertigo episodes to progressive bilateral vestibular loss, tinnitus, and aural fullness—symptoms like MD [8,9]. Visual oscillations may also occur and progress with age [7]. Due to these similarities, the COCH gene has been studied in MD patients from various populations, but no significant associations have been found. Sanchez et al. showed that known mutations in exons 4 and 5 of COCH have a low prevalence in MD [58] and Usami et al. concluded that COCH mutations are not a major cause of typical MD [59]. In a Belgian family with DFNA9, Fransen et al. identified the P51S mutation in COCH, associated with hearing loss and vertigo [60]; however, it remains unclear whether these cases can be considered true MD according to the studies by Verstreken and Frykholm [61,62].

This discrepancy can be explained, at least in part, by selection bias in family studies and by genetic differences between populations, suggesting that the association between DFNA9 and Ménière-like symptoms may be specific to certain genetic backgrounds. Morrison and Johnson proposed a distinct genetic locus for familial MD on chromosome 14q, separate from COCH. In their series, linkage data indicated a partially overlapping region with COCH, but mutational analysis did not identify any known or novel mutations, leading to the conclusion that the familial locus does not correspond to COCH, without proposing an alternative candidate gene [1,7].

Thus, DFNA9 and MD are considered separate entities: DFNA9 is characterized by early-onset high-frequency hearing loss with progressive vertigo, whereas MD has a later onset and initially affects low frequencies. Furthermore, while the temporal bones of DFNA9 patients may show EH, the distinctive feature of DFNA9 is the presence of microfibrillar deposits in the stria vascularis, which are absent in MD [9].

### 2.5. HLA Genes in Ménière’s Disease

The major histocompatibility complex (MHC) is a set of genes that encode histocompatibility antigens in animals. In humans, this complex is known as the human leukocyte antigen (HLA). HLA genes are located on chromosome 6, region 6p21.31, and exhibit a high degree of polymorphism, being closely linked to immune response mechanisms [3].

These genes are grouped into three classes: Class I genes (HLA-A, -B, -C, -E, and -F) are expressed on the surface of all nucleated cells; Class II genes (HLA-DR, -DQ, and -DP) are mainly found on antigen-presenting cells; and Class III genes include components involved in the complement system, tumor necrosis factor (TNF), heat shock proteins (HSPs), and transcription factors, located between the Class I and Class II regions. Numerous studies have shown that Class II HLA genes play a central role in the development of most autoimmune diseases. Additionally, MICA (MHC class I chain-related gene A), considered an atypical Class I gene, interacts with specific receptors on immune cells, acting as a ligand and influencing immune responses. Thus, MICA may play an important role in the pathogenesis of autoimmune diseases [14]. Given that immunological factors appear to contribute significantly to the development of MD, multiple studies have investigated the relationship between the HLA complex and this condition [3,9].

Morrison et al. observed that the predominant haplotype in their cohort of 70 patients was A3-B7-CW7-DR2, hypothesizing that if a single mutation underlay susceptibility to MD, it would likely occur on the chromosome carrying CW7, given the consistent association of HLA-A3 and HLA-B7 with this antigen. Based on this correlation and the known genetic distances between the three HLA genes, the authors estimated that the locus involved in MD predisposition would be located approximately 350 kb from the HLA-C locus, in the short arm region of chromosome 6 [1].

Several studies have examined these associations, but the results have often been inconsistent. Xenellis et al. were among the first to report a link between the HLA-Cw07 allele and MD in 41 British patients [63]. Two studies replicated the association with HLA-Cw07, showing a significantly higher frequency of this allele in MD patients compared with controls or patients with other inner ear disorders, suggesting a possible predisposing role [64,65]. Studies supporting increased susceptibility were primarily conducted in European populations; therefore, expanding study cohorts to other populations is necessary to determine whether similar associations are observed across different ethnic groups. Koyama et al. identified an association with the DRB1*1602 allele in a Japanese population [66]. Lopez-Escamez et al., studying a larger sample from the Spanish population, did not find significant differences in the frequency of HLA-Cw07 or the DRB1*1602 allele, suggesting that previously reported immunological alterations may represent only epiphenomena of inner ear pathology [64]. However, the same group later observed an association between HLA-DRB1*1101 and bilateral MD, indicating that certain alleles may be involved only in specific or more severe forms of the disease across different populations [67].

Khorsandi et al. reported an association between defined MD and the HLA-Cw04 allele, reinforcing the idea that certain HLA variants may contribute to individual susceptibility to MD [68]. Koyama et al. did not find a significant correlation between HLA-Cw04 and MD in an Asian population [66]. However, the study by Lopez-Escamez et al. reported an association between the two in an Asian population [64]. The association between HLA-Cw*04 and MD remains controversial, as study results have been inconsistent across different populations. This lack of consistent correlation suggests that the effect of this allele is population-dependent and may be influenced by linkage disequilibrium with other HLA genes, differences in allele frequencies, or distinct environmental factors. These findings support the hypothesis of an immunogenetic role for HLA-Cw*04 in disease susceptibility, possibly by facilitating a persistent cytotoxic immune response in the inner ear [9]. Regarding HLA-DRB1*1201, HLA-DRB1*0405 [69], and HLA-DRB1*15, HLA-Cw*0303 [70], Koo et al. and Yeo et al. observed potential MD susceptibility associated with these variants in the Korean populations studied. These studies have not been replicated in other populations, raising the question of whether these correlations are specific to Asian populations or if potential associations also exist in European populations.

The observation that certain HLA alleles, such as HLA-DRB1*09 [71], HLA-B44 [70], HLA-DRB1*13 [69] and HLA-DR2 [64], have been reported to exert a slightly protective effect against MD is consistent with the central role of the HLA complex in regulating immune responses. HLA does not cause the disease directly; rather, it shapes how the immune system recognizes and responds to antigens, which can lead to either susceptibility or protection. A possible mechanism underlying this protective effect is more tightly controlled antigen presentation [69]. Certain HLA alleles may present inner ear antigens or microbial antigens in a way that promotes immune tolerance, thereby reducing the risk of activation of autoreactive T lymphocytes. In this context, alleles considered protective may limit the initiation of chronic inflammatory responses in the endolymphatic sac, preventing epithelial damage and the development of EH [3]. Another important mechanism is related to the polarization of the immune response. Some HLA variants are associated with a more balanced immune profile, characterized by reduced production of proinflammatory cytokines and an enhanced capacity for immune regulation. Thus, in the presence of triggering factors such as viral infections or oxidative stress, carriers of these alleles may develop a transient and controlled inflammatory response without progression to persistent inflammation and endolymphatic dysfunction [9].

To date, association studies between the HLA complex and MD have failed to provide reproducible results for all proposed variants, and further research is required to confirm these findings. One of the major issues identified in the literature is the lack of consistency across the populations studied, which can be explained by genetic differences and variations in HLA expression among different ethnic groups. These particularities may influence disease susceptibility and the regulation of immune responses, which could explain why some studies have found significant associations while others have been unable to replicate the results [3,9].

Another important limitation is the small sample size included in most studies, which reduces the statistical power of analyses and increases the risk of false-positive or false-negative results. In the absence of multicenter studies with large, well-characterized cohorts, it is difficult to establish a solid causal link between specific HLA gene variants and MD pathogenesis. Furthermore, it is possible that the role of HLA genes is not directly causal but rather influences susceptibility to MD through complex immunological mechanisms, such as abnormal T cell activation, altered antigen presentation, or inadequate regulation of inflammatory responses [3].

These processes may contribute to the development of chronic inflammation in the inner ear and to the fluid and ionic dysfunctions observed in EH. Therefore, the question remains whether mutations or polymorphisms in HLA genes constitute direct causal factors for MD or merely serve as genetic markers associated with immunological predisposition. Clarifying this issue requires large-scale studies conducted on ethnically diverse cohorts that integrate genetic, epigenetic, and immunological analyses. Only through such integrated approaches can it be determined whether HLA has a direct pathogenic role or simply participates in a broader multifactorial context of the disease [3,9].

In conclusion, although the data on HLA and MD are still inconclusive and sometimes contradictory, they suggest that certain alleles may influence disease risk, and their effect may depend on population, type of MD (sporadic or bilateral), and other genetic or environmental factors. All HLA genes proposed in studies as having, or not having, an association with MD are summarized in Table 2.

### 2.6. Genes Related to Aquaporins and Ion Channels in Ménière’s Disease

In the inner ear, aquaporins (AQPs), subtypes 1–5, 7, and 9, play a key role in maintaining water and ion balance. These specialized proteins facilitate the transport of water and small molecules, such as glycerol. Additionally, potassium channels from the KCNE family contribute significantly to the transmembrane movement of ions and water. Given that the fundamental pathological mechanism of MD is endolymphatic hydrops, it is reasonable to assume that genes encoding these proteins are involved in the development of the disease. However, studies analyzing polymorphisms in AQP and KCNE genes have yielded contradictory results, varying across populations [3,13].

Beyond these proteins, other cellular mechanisms are critical for maintaining endolymph composition stability. Na^+^, K^+^-ATPase and the sodium-calcium exchanger play fundamental roles in ionic regulation, and their dysfunction may contribute to abnormal fluid accumulation [11,12,13]. In addition, adducin (ADD), a cytoskeletal protein composed of α, β, and γ subunits (encoded by ADD1, ADD2, and ADD3), has relevance [3].

Along the same lines, SIK1 (Salt-Inducible Kinase 1), which encodes salt-inducible kinase 1 and modulates Na^+^, K^+^-ATPase activity, as well as SLC8A1, responsible for the sodium-calcium exchanger, have been associated with MD. According to Teggi et al., polymorphisms in ADD1, SIK1, and SLC8A1 appear to contribute to disease susceptibility [72,73]. These findings support the hypothesis that ionic transport disturbances and cellular homeostasis imbalances play a central role in the onset and progression of MD [3].

#### 2.6.1. Aquaporins

A major area of interest in MD research is mutations in the aquaporin gene family. Aquaporins are transmembrane proteins essential for the transport of water and small dissolved molecules, playing an important role in maintaining the fluid balance of the endolymphatic sac and, consequently, of the inner ear. To date, several AQP subtypes have been identified in this region, including AQP1, AQP2, AQP3, AQP4, and AQP5 [9,74]. Mutations in these genes have been proposed as contributing factors to altered osmotic balance in endolymph, potentially leading to the development of EH [75]. Among the aquaporins, AQP1–5 are the most studied in the context of MD, with particular emphasis on AQP2, the only isoform hormonally regulated by vasopressin or antidiuretic hormone (ADH/AVP). AQP2 plays a central role in controlling water flow in the inner ear and in the precise regulation of water reabsorption, thereby contributing to fluid homeostasis [12,76].

Mhatre et al. tested the hypothesis that AQP2 gene mutations might be involved in MD by analyzing 12 unrelated patients [77]. The results did not reveal any sequence alterations in this gene. Subsequently, two other studies expanded the analysis to additional members of the aquaporin family (AQP1–AQP4), yet these also did not identify causal mutations associated with the disease [75,78]. However, in the study by Candreia et al., a partial correlation with MD was observed for AQP3 homozygous c.105G>C. A known variant of the AQP5 gene (AQP5–1364 A/C), although influencing the transcription of the protein involved in cellular homeostasis, does not appear to play a significant role in regulating inner ear fluid balance in patients with MD [79]. In a study by Nishio et al. conducted in a Japanese population, AQP5 rs3736309 showed a potential association with MD [80]. These data suggest that, although aquaporins play a physiologically essential role in regulating inner ear fluids, direct mutations in their genes do not appear to be a frequent cause of MD. Nevertheless, it cannot be excluded that variations in expression or interactions with other genes involved in ionic homeostasis could indirectly contribute to susceptibility to this condition [9].

Exonic sequencing performed in the study initiated by Lopes et al. identified SNP rs426496 in exon 2 of AQP2 and SNP rs591810 in exon 1 of AQP3. These results are consistent with those reported by Candreia et al. [78], both regarding the molecular technology used and the polymorphisms identified. Like that study, no significant differences were observed between patient and control groups, suggesting that these variants cannot be considered direct susceptibility markers for MD in the analyzed population. However, unlike previous works, Lopes’ study emphasizes the importance of correlating these genetic variants with the clinical profile of patients, an aspect not investigated in earlier studies and essential for understanding disease heterogeneity [12].

One of the central observations is the identified association between SNP rs426496 in AQP2, the presence of tinnitus at disease onset, and changes detected via electronystagmography (ENG), as reported by Lopes et al. [12]. This correlation suggests that certain genetic variations may directly influence the manifestation of vestibular dysfunction, and clinical cases with ENG alterations (whether hypofunction, hyperreflexia, or positional nystagmus) acquire additional diagnostic relevance considering this finding. Beyond their immediate clinical significance, these observations reinforce the idea that functional parameters of the labyrinth may subtly reflect molecular aspects of endolymphatic homeostasis [12]. All variants cited in the literature are presented in Table 2.

This discrepancy between studies highlights that AQPs are functionally plausible candidate genes, but their role in genetic susceptibility to MD remains unclear and is likely multifactorial. Their effect may be more physiological, influencing fluid homeostasis, rather than strictly genetic, and the identification of variants with significant clinical impact will require large, reproducible cohort studies.

#### 2.6.2. KCNE1 and KCNE3

Genes involved in the regulation of aquaporins and K^+^ channels have frequently been selected for association studies due to their direct role in the dynamics of labyrinthine fluids. Both experimental data and association studies suggest that these genes may contribute significantly to the etiology of MD [12,81]. Histological studies have shown that these channels exhibit differentiated tonotopic expression (i.e., their expression levels vary between the basal and apical portions of the cochlea, reflecting functional specialization across auditory frequencies) [8,82].

KCNE1 is predominantly expressed in the stria vascularis and in the epithelial cells of the endolymphatic duct, ensuring the regulation of K^+^ flow in the endolymph. Dysfunction of this subunit may compromise the endocochlear potential and ionic balance, mechanisms considered relevant for the development of EH [10]. KCNE3 is expressed in the epithelium of the endolymphatic sac and is involved in the transepithelial regulation of ions and water. Its role in maintaining fluid homeostasis suggests a mechanism by which dysfunction or genetic variants could influence the progression of EH and the auditory and vestibular manifestations of the disease [9].

Analysis of the KCNE1 gene revealed three SNPs located in exon 1: rs1805127, rs1805128, and rs17173510. Among these, rs1805127 is the most well-documented in the literature, reported by Doi et al. [10], Campbell et al. [83], Lopes et al. [12] and Hietikko et al. [84]. The study by Lopes et al., conducted in a Brazilian population, found a strong correlation of rs1805127 with MD, compared to the other SNPs, which showed lower statistical significance (rs1805128, rs17173510) [12]. Hietikko et al. identified significant differences in allele distribution for KCNE1 rs1805127 between patients with MD and controls, suggesting that this SNP may represent a genetic susceptibility factor in that population, especially for sporadic forms of the disease. The same study also demonstrated a significant association between SNP genotypes and disease presence in the Finnish population studied. Furthermore, it identified four novel sequence variations in KCNE1 in four patients (three with sporadic forms and one with a familial form), suggesting that these mutations could be disease-causing, although confirmation requires further studies in larger cohorts [84].

Based on the hypothesis that dysfunction of these channels could influence MD development, Doi et al. reported that certain allelic variants of KCNE1 (rs1805127) and KCNE3 (198T/C) showed significantly different frequencies in an affected Japanese population, suggesting a possible role as susceptibility genes for sporadic MD [10]. However, the allele distribution in the control group did not meet Hardy–Weinberg equilibrium, indicating either inadequate selection of the reference group or genotyping errors [7]. Ideally, the control group should exhibit a stable allele distribution, with significant deviations observed only in the disease cohort. In the absence of this essential criterion, the associations proposed by Doi et al. were invalidated. Conversely, the study conducted by Campbell et al. found no association in a Caucasian population and could not confirm Doi’s findings for either variant [83]. A recent meta-analysis also showed that KCNE1 rs1805127 and KCNE3 rs2270676 do not have a significant association with MD risk [85]. Similar results were reported by Vrabec et al., who failed to confirm the association of KCNE1 rs1805127 [86].

In a recent study, Dai et al. observed a correlation between the KCNE1 653 C/T variant and MD in a Chinese population [13]. The same study also found that KCNE3 polymorphisms were key for patients with FMD, whereas KCNE1 was more relevant for the onset of SMD. This contrast between datasets supports the notion of a population-specific effect, a phenomenon commonly observed in the genetics of complex diseases. KCNE1 polymorphisms may influence susceptibility to MD within a particular genetic context, suggesting that future studies should include diverse populations to clarify whether these variants are universal risk factors or specific markers for certain ethnic groups [12].

The rs1805127 variant of KCNE1 has also been reported as associated with nephropathy in Brazilian patients with MD, indicating potential links between potassium channel dysfunction and renal impairment. Although the number of such cases is limited, the spectrum of renal involvement (ranging from nephrolithiasis to forms of glomerulosclerosis) raises the question of possible shared mechanisms between ion channel dysfunction in the inner ear and renal susceptibility. This connection aligns with observations in other pathologies where ion channel proteins exhibit multi-organ expression and physiological roles [8,12]. Thus, although KCNE1 and KCNE3 represent biologically plausible candidate genes for MD susceptibility, current data remain inconsistent and incomplete. This highlights the need for further studies in diverse ethnic groups with larger sample sizes to clarify whether these genes are truly involved in the pathogenesis of EH [3,13]. All variants cited in the literature are presented in Table 2.

#### 2.6.3. Adducin

Adducin is a heterodimeric cytoskeletal protein composed of three subunits (alpha, beta, and gamma), encoded by the ADD1, ADD2, and ADD3 genes. ADD1 is involved in the formation and stabilization of cytoskeletal complexes and influences the activity of ion pumps and transepithelial channels. Genetic variants or polymorphisms of this gene may alter ionic balance and contribute to endolymph accumulation, increasing susceptibility to the characteristic symptoms of MD. ADD2 acts in concert with ADD1 to maintain cytoskeletal structure and support the function of ion channels and pumps. This protein is essential for the stability of epithelial cell membranes in the inner ear and may partially explain the phenotypic variability observed among patients. ADD3 plays a role in the final organization of spectrin–actin complexes and in connecting them to specific cellular membranes. ADD3 function influences the permeability of the endolymphatic sac epithelium and the efficiency of transepithelial ion transport, which may promote the development of EH [72].

The ADD1 Gly460Trp polymorphism has previously been associated with salt-sensitive hypertension and increased Na^+^/K^+^ pump activity in transfected cells, suggesting an impact on ionic balance [87]. The frequency of the ADD1 Trp allele (rs4961) was significantly higher in patients with MD compared to healthy controls, indicating that this variant may contribute to individual susceptibility to the disease. Teggi et al. did not identify significant differences in genotype distribution for ADD2 and ADD3 polymorphisms [72].

Polymorphisms of the ADD1 gene, particularly rs4961, are associated with salt-sensitive hypertension due to the role of this gene in regulating renal sodium reabsorption. Individuals carrying these variants exhibit increased sodium retention and expanded blood volume, which can lead to elevated blood pressure, especially in the context of a high-salt diet [72].

This hemodynamic effect is relevant for the inner ear, where the endolymphatic sac regulates fluid and ion homeostasis within the membranous labyrinth. Increased blood pressure and microcirculatory changes associated with ADD1 polymorphisms can impair cochleo-vestibular perfusion, induce oxidative stress, and promote local inflammation, thereby compromising the function of epithelial transport mechanisms in the endolymphatic sac. These disturbances facilitate abnormal endolymph accumulation, contributing to the development of endolymphatic hydrops [3,11].

The influence of ADD1 polymorphisms clearly demonstrates a gene–environment interaction, as individuals without the risk alleles generally tolerate a high-salt intake without developing endolymphatic imbalance, whereas carriers of these variants are more susceptible to hydrops under the same dietary conditions. Although ADD1 polymorphisms alone are not sufficient to cause MD, they create a physiological environment in which other factors, such as chronic inflammation or oxidative stress, can trigger clinical manifestations of the disease, including vertigo attacks, hearing loss, and tinnitus [11,72].

α-Adducin is a cytoskeletal protein involved in stabilizing the cell membrane and, critically, in regulating Na^+^/K^+^-ATPase activity in epithelial tissues. The Gly460Trp variant is known to increase the activity of this pump, resulting in enhanced sodium and water reabsorption in various epithelial tissues. Extrapolating this mechanism to the inner ear, increased ionic transport activity in the labyrinthine epithelia or the endolymphatic sac may disrupt the osmotic balance of the endolymph, promoting fluid retention. This subtle but persistent alteration of ionic homeostasis can lead to progressive endolymph accumulation and the development of EH, representing a plausible molecular mechanism through which the ADD1 Gly460Trp variant contributes to susceptibility to MD, particularly in forms influenced by hydroelectrolytic regulation [72]. In contrast, ADD2 and ADD3 primarily act as modulatory subunits, with expression more restricted to specific cell types and a lesser impact on the overall control of ionic fluxes. Moreover, known variants of ADD2 and ADD3 have not been associated with significant functional changes in ionic transport or fluid volume, which are essential in the pathophysiology of EH. Thus, in the context of the inner ear, ADD1 occupies a functional node critical for regulating endolymphatic homeostasis, whereas ADD2 and ADD3 do not appear to directly influence these processes, explaining the absence of a clear association with MD [72,87].

These data support the hypothesis that increased Na^+^/K^+^-ATPase activity, influenced by the ADD1 polymorphism, could lead to hyperosmolarity in the endolymph, potentially promoting the development of EH by disrupting the osmotic balance and fluid volume of the endolymphatic compartment. However, this association has not been replicated in other independent cohorts, which limits the validity of these conclusions and highlights the need for larger replication studies [9,84]. All variants cited in the literature are reported in Table 2.

#### 2.6.4. Antiquin

Another candidate gene that has been investigated is antiquin (ATQ), due to its structural homology with the plant protein 26 g, which is involved in ion channel function and fluid balance regulation [88]. Based on this similarity, it has been hypothesized that antiquin may participate in the regulation of endolymphatic homeostasis. However, comparative studies between patients and control groups have not revealed significant differences, and the association remains unconfirmed. In the context of MD, ATQ1 is considered a candidate gene because cytoskeletal dysfunction and disruptions in transepithelial transport can affect endolymph homeostasis. It is hypothesized that rare genetic variants or polymorphisms in ATQ1 could influence susceptibility to MD by altering cellular responses to oxidative stress, electrolyte imbalances, or inflammation, but these theories are not yet supported by studies and need to be investigated in diverse populations for validation [8].

### 2.7. Genes Related to Immunity, Inflammation, and Oxidative Stress in Ménière’s Disease

In recent years, genetic variants have been identified in genes involved in the regulation of inflammation in patients with SMD, supporting the hypothesis that certain molecular predispositions may favor the establishment of persistent inflammatory activation [8]. However, the diversity of clinical phenotypes in MD complicates the establishment of firm genetic criteria for defining subgroups. Patients with a family history or associated autoimmune diseases may represent a distinct subset, yet the genetic delineation of the autoinflammatory form of MD has not been established [20,89]. A case of refractory MD has been documented, showing features suggestive of an autoinflammatory process both phenotypically and genotypically, in which high-dose local steroid administration at the endolymphatic sac produced a robust improvement in symptoms (an observation that underscores the relevance of the inflammatory mechanism in this pathology) [90].

Beyond the HLA complex, a wide range of other genes involved in immunological, inflammatory, and oxidative stress processes have been investigated to establish potential links with MD [3,91]. Among the genes involved in inflammation regulation associated with MD are NFKB1 of the NF-κB pathway [16], the histamine H4 receptor gene (HRH4) [92], which mediates the regulation of proinflammatory factors, and the RANTES gene, linked to inflammatory diseases [93]. For NFKB1 rs3774937 and NFKB1 rs4648011, Cabrera et al. identified a role in the progression of unilateral forms of MD in a Spanish population, particularly regarding unilateral hearing loss [16]. For HRH4, Qin et al. observed a strong association with vertigo in MD in a Chinese population [92]. For RANTES-403A, a protective effect in MD was observed in an Italian male population [93]. Lopez-Escamez et al. reported that the 1858 C/T polymorphism of the PTPN22 gene, which encodes a non-receptor tyrosine phosphatase, is associated with an increased risk of developing MD in the Hispanic population. This gene plays a key role in regulating T cell activation, and the mentioned variant reduces their functional capacity, supporting the hypothesis that autoimmunity may represent a major etiologic factor in disease onset [3,94].

Studies conducted on the Japanese population have identified associations between susceptibility to MD and several genetic polymorphisms, including in the interleukin-1 alpha (IL-1A) gene [95], the macrophage migration inhibitory factor (MIF-173 G/C) [96,97] and the heat shock protein HSPA1A [98]. Based on the hypothesis that the immune system plays a central role in the pathogenesis of MD, researchers have focused on the MIF gene, which is involved in regulating the immune response. MIF is an essential proinflammatory cytokine, involved in macrophage migration and the regulation of blood–brain barrier permeability, making this gene a biologically relevant candidate for investigating the immunological mechanisms involved in MD. Yazdani et al. investigated the association of the MIF-173 G/C polymorphism in an Iranian MD population, suggesting that this variant could contribute to MD susceptibility [97].

Additionally, Gázquez et al. studied a Spanish cohort and identified a significant association between MD and the MIF allele containing five CATT repeats, while the same association was not observed in the American cohort of the study [96]. This suggests possible abnormal macrophage activation and a role for proinflammatory cytokine–mediated neuroinflammation in disease progression [96]. Further studies have highlighted that patients with MD exhibit a distinct gene expression and cytokine profile compared to those with vestibular migraine, which could indicate the involvement of allergic or inflammatory processes as triggering factors in certain cases [8,99]. These findings suggest that the effect of MIF polymorphisms on MD susceptibility may be influenced by ethnic or population-specific genetic factors [9].

Starting from the hypothesis that MD could be influenced or triggered by psychological stress, Kawaguchi et al. analyzed two polymorphisms in the HSPA1A gene, which encodes the 70 kD heat shock protein involved in the cellular stress response. The study identified the SNP 190 G/C (rs1043618) as significantly associated with MD, while the other SNP (rs1008438) showed no association [98]. Interestingly, the same SNPs have also been implicated in other conditions, such as stroke, cardiovascular diseases, open-angle glaucoma, Parkinson’s disease, and noise-induced hearing loss, yet there is no direct clinical correlation between these diseases and MD. This suggests that the effect of HSPA1A on MD is not direct but may act in conjunction with other as-yet-unknown genetic or environmental factors. Further studies are needed to determine whether dysfunction of this protein could be a primary cause of MD or a predisposing factor in the context of additional conditions [7,9,100,101].

Supporting the autoinflammatory hypothesis, elevated concentrations of the cytokine IL-1β have been observed in a subset of patients without detectable autoantibodies, suggesting direct activation of inflammatory mechanisms in the absence of a classical autoimmune process [95]. Additionally, Frejo et al. reported increased levels of the cytokines IL-1β, IL-1RA, TNFα, and IL-6 in approximately 21% of patients with MD, supporting the hypothesis that these individuals may represent a subset of autoinflammatory disorders [8,102].

Regarding CTLA4, TNF-α, and IFN-γ, studies conducted in the Spanish and American populations did not find a clear association with MD [94,96]. Studies investigating specific genes, NOS1 and NOS2A [96,103], also failed to demonstrate a consistent relationship between these genetic variants and the risk of MD. Additionally, Toll-like receptors, such as TLR10, which are part of the innate immune system and involved in pathogen recognition, have also been analyzed. The rs11096955 variant of the TLR10 gene was correlated with a more favorable auditory prognosis in Spanish and Italian patients, indicating that not all genetic alterations have negative consequences; some may positively influence clinical outcomes by modulating the nonspecific immune response [3,15]. These findings suggest that inflammation and cellular stress responses play a significant role in the pathogenesis of the disease, possibly through excessive activation of proinflammatory pathways and disruption of cellular protective mechanisms [3].

Beyond the well-known, widely discussed, or previously studied genes, new promising avenues are emerging that both shed light on the disease process and complicate some of the previously understood mechanisms. The study by Zou et al. is extensive and involves genetic variants related to inflammatory and autoimmune processes detected in patients with sporadic MD in a Chinese population. One example from the study is the MEFV gene, which encodes the pyrin protein. Pyrin is an important regulator of IL-1β–mediated inflammation. Dysregulation of this pathway may influence inflammatory processes in the inner ear [91]. Moreover, recent studies have shown that exposure of epithelial cells from the human endolymphatic sac to IL-1β leads to reduced expression of epithelial sodium channels and associated currents, which may impair fluid absorption at this site [11]. Considering that MEFV mutations are well-known markers of autoinflammatory diseases and have also been described in certain autoimmune pathologies, it is plausible that inflammatory dysregulation associated with these variants may also influence the progression of MD [91,104,105].

COL7A1 is a gene involved in the synthesis of type VII collagen, a protein essential for the structural integrity of tissues. Mutations in this gene are classically known to cause dystrophic epidermolysis bullosa, a disease characterized by chronic inflammation and well-documented autoimmune mechanisms [91]. Beyond this well-established role, certain genetic alterations in COL7A1 may also have functional implications at the auditory level. Specifically, the genomic region of COL7A1 partially overlaps with that of the TMIE gene, which is essential for the process of mechanoelectrical transduction in cochlear hair cells. Alterations affecting this shared region have previously been described in a case of bilateral auditory neuropathy [106]. In the context of the study conducted by Zou et al., it is plausible that the identified mutation, which introduces a premature stop codon in the TMIE gene, leads to a nonfunctional protein. However, the clinical manifestations of the analyzed patients do not fully correspond to the classical phenotype of auditory neuropathy. This discrepancy suggests the possible presence of comorbidity, in which MD coexists with an auditory impairment within the auditory neuropathy spectrum, reflecting the complexity and heterogeneity of the genetic mechanisms involved [91].

Immune dysfunction can affect inner ear homeostasis through chronic activation of the inflammatory response in the endolymphatic sac, a structure essential for regulating endolymph volume and composition. Persistent inflammation may increase vascular permeability, promoting fluid extravasation and disrupting ionic balance, which leads to abnormal endolymph accumulation and the development of endolymphatic hydrops. Immune cells recruited within the endolymphatic sac can secrete proinflammatory cytokines and cytotoxic factors that compromise epithelial integrity and ion channel function. This epithelial disruption interferes with endolymph resorption mechanisms and can generate oscillations in endolymphatic pressure, explaining the episodic nature of symptoms in Ménière’s disease. The immune process can be triggered or amplified by autoimmunity, allergies, or latent viral infections, which induce molecular mimicry or inappropriate local immune activation. The interaction between genetic factors and immune activation contributes to individual susceptibility and to the phenotypic variability observed among patients [2,3,91].

COLEC11, which encodes the CL-K1 component of the collectin family, plays an essential role in the molecular recognition of pathogens, host defense, and tissue homeostasis. Mutations in this gene can lead to 3M syndrome, which is characterized by craniofacial abnormalities, developmental disorders, and multisystem dysfunction. Although the clinical phenotype differs from MD, the impact of these mutations on immune recognition mechanisms may indirectly contribute to inflammatory susceptibility of the inner ear [91].

The ADA gene, which encodes adenosine deaminase, plays a central role in the purine salvage pathway. Severe defects in this enzyme lead to the accumulation of toxic metabolites such as 2′-deoxyadenosine, with devastating effects on lymphocytes, resulting in severe combined immunodeficiency. Interestingly, ADA deficiency has also been associated with sensorineural hearing loss [91,107]. ADA-deficient murine models (ADA−/−) develop marked damage to cochlear hair cells and immune dysfunction; these manifestations can be ameliorated by enzyme replacement therapy, suggesting a direct link between purine metabolism and the homeostasis of auditory structures [91].

RAG2 plays an important role in the diversity of lymphocyte receptors. Mutations in RAG2 can severely impair lymphocyte maturation, leading to a predisposition to autoimmune diseases. Similarly, mutations in BLM, which encodes a helicase essential for DNA stability, cause Bloom syndrome, a condition associated with significant immunological disturbances. The presence of such variants in patients with MD could contribute to a vulnerable immunological background that favors the development of chronic inflammation [91,108].

RNF31 is an essential component of the linear ubiquitination complex, with a direct role in NF-κB signaling, cell survival, and regulation of inflammation. Variants in this gene have been linked to immunodeficiency, autoinflammation, and other severe disorders such as amylopectinosis or lymphangiectasia [109]. Additionally, FAT4, a gene encoding a massive protein involved in cell polarity and tumor suppression, can, through its mutations, cause complex neuronal defects and Van Maldergem syndrome, characterized by distinctive facial features, central nervous system abnormalities, and renal or skeletal malformations [110]. Although these pathologies are rare and more severe than MD, their involvement in essential cellular processes suggests that heterozygous variants could modulate tissue vulnerability [91].

The analysis of rare and potentially pathogenic genetic variants identified in patients included in the studies highlighted several mutations that may have biological relevance for understanding the mechanisms involved in MD. Among these, TLR3 is of particular importance, as it is a receptor known for its central role in the pathogenesis of immune-mediated lupus nephritis, where it promotes sustained type I interferon signaling [111]. Additionally, the variant identified in RAB27A, a gene involved in the regulation of exosome secretion and in directing lytic granules toward immune synapses, suggests potential disturbances in intercellular communication and immune-mediated cytotoxicity mechanisms [91,112].

Another set of variants was identified in genes belonging to the FANC family, which encode proteins involved in the repair of DNA interstrand crosslinks. Under normal conditions, these proteins contribute to the maintenance of genomic integrity and to the proper functioning of the immune system. Mutations in these genes are responsible for Fanconi Anemia, a complex disorder characterized by congenital abnormalities, progressive hematopoietic failure, and an increased predisposition to cancer. The presence of such variants in the analyzed patients with SMD may suggest an intrinsic vulnerability to cellular stress or organellar dysfunction, factors that may also indirectly influence inner ear pathology [91,113].

Another gene of interest is LPIN2, whose protein product, lipin 2, functions as a magnesium-dependent, membrane-associated phosphatase. Recessive mutations that reduce the function of this protein have been implicated in dysregulation of innate immune responses, leading to severe systemic inflammation and osteomyelitis in Majeed syndrome. Similarly, mutations in the NBAS gene, which encodes a protein essential for retrograde transport between the Golgi apparatus and the endoplasmic reticulum, can disrupt vesicular trafficking and induce abnormal formation of large vesicles at endoplasmic reticulum exit sites. These disturbances have been associated with cellular stress and amplification of inflammatory processes at the level of the endoplasmic reticulum [91,114].

Overall, the presence of such mutations in the investigated patients supports the idea that SMD may represent, at least in some cases, the expression of a susceptible genetic background in which multiple immune pathways are subtly altered. The complex interaction between these variants and the inflammatory microenvironment of the inner ear may contribute to the diversity of clinical manifestations, underscoring the need for extensive genetic studies to clarify these connections [91].

Although these pathologies do not directly overlap with MD, they confirm that many of the rare variants detected in the examined patients with MD are linked to fundamental immune mechanisms, suggesting that inflammatory predisposition or subtle immune dysregulation may constitute a common and relevant background for the phenotypic expression of the disease [91]. Overall, the mutations identified in the analyzed genes appear to outline a complex picture of how immune dysregulation can intervene at different stages of the biological response. Whether involving mechanisms that ensure genomic integrity and stability, processes of post-translational modification and intracellular protein trafficking, or the functioning of the exosomal pathway, each genetic variant has the potential to alter a critical segment of immune architecture. Although the variants detected in patients with MD in the study conducted by Zou et al. are not identical to those identified in other diseases, these variants are deleterious to the gene products and may induce pathological changes in affected individuals [91].

Interestingly, some of these variants intersect with molecular pathways already implicated in auditory disorders. For example, mutations affecting RNF31, an essential component of the linear ubiquitination process required for activation of NF-κB signaling, are conceptually related to observations showing that regulatory variants in NFKB1 can influence hearing outcomes in patients with MD and in those with unilateral sensorineural hearing loss. Along similar lines, the TLR3 pathway (an indispensable component of the innate immune response) has already been associated with the defense mechanisms of the human endolymphatic sac, suggesting that the sensitivity of this structure to inflammatory stimuli may have major functional consequences [16,91,115].

Given the intersection of these mechanisms, it is unlikely that a single, definitive set of mutations specific to MD can be identified, particularly since autoimmune and autoinflammatory processes (far from being confined to a single organ) can simultaneously affect multiple biological systems. Nevertheless, the presence of these deleterious genetic variations may increase the susceptibility of certain individuals to developing the disease when other triggering factors, whether environmental or biological, act cumulatively. Thus, what emerges is not a single unique cause, but rather a vulnerable background in which genetic predispositions and inflammatory contexts converge, each amplifying the likelihood of MD onset [3,17,91].

Patients with SMD may carry a broad spectrum of genetic variants involved in the modulation and coordination of the immune response. The presence of these genetic alterations may create an immunologically vulnerable background, predisposing individuals to the initiation of inflammatory processes through both autoimmune and autoinflammatory mechanisms, particularly when the organism is exposed to stressors. In such a context, the development of EH may represent the outcome of the interaction between genetic predisposition and biological or psychophysiological stimuli arising from the internal or external environment. However, to confirm these hypotheses with certainty and to robustly assess how these genetic variants contribute to disease onset and progression, large patient cohorts are required. Only studies conducted in large populations can reproduce and validate current findings, thereby providing a clear perspective on the true role played by these genetic variants in the pathogenesis of MD [3,17,91]. All the genes discussed in this chapter are presented in Table 2, with additional explanations.

**Table 2:** Genes proposed in studies as being involved in the development of MD.

	The Gene Involved	Studied Population	Correlation with MD	The Study and the Authors Who Mention It	Remarks
HLA genes	MICA*A.4	Spanish population	Protective effect for MD	(Gazquez et al., 2012) [14] (Dai et al., 2023) [3]	Mechanistically, the hypothetical protective effect of MICA*A.4 may be related to the ability of the molecules encoded by this allele to modulate innate immune responses. This could limit the overactivation of NK cells and T lymphocytes in the inner ear, thereby preventing inflammation of the endolymphatic sac and the development of EH. However, it is important to emphasize that this association has been observed in only one study and has not been replicated in other cohorts. The studied cohorts were relatively small and may have been influenced by population stratification or linkage disequilibrium in the HLA/MICA region. Without independent replication, the protective role of the MICA*A.4 allele remains a preliminary hypothesis and cannot be considered a valid protective marker in clinical or genetic practice [3,52].
	HLA-DRB1*09	Chinese population	Protective effect for MD	(Meng et al., 2001) [71] (Dai et al., 2023) [3]	It is essential to emphasize that this protective effect is neither absolute nor universal. HLA-DRB1*09*, *HLA-B44*, *HLA-DRB1*13, and HLA-DR2 may be considered factors of relative protection rather than deterministic factors. They appear to reduce the likelihood of disease development or severity through mechanisms involving immune tolerance, inflammation control, and limitation of cytotoxicity. However, this protective effect is modest, population-dependent, and context-specific, and its confirmation requires multicenter studies, haplotype-based analyses, and functional correlations. From a clinical perspective, these alleles are more valuable for understanding the immunological pathogenesis of MD than as individual predictive markers [3].
	HLA-B44	South Korean population	Protective effect for MD	(Yeo et al., 2002) [70] (Dai et al., 2023) [3]	
	HLA-DRB1*13	South Korean population	Protective effect for MD	(Koo et al., 2003) [69] (Dai et al., 2023) [3]	
	HLA-DR2	American population	Protective effect for MD	(López-Escámez et al., 2002) [64] (Dai et al., 2023) [3]	
	HLA-DRB1*1101	Spanish population	Not correlated with bilateral MD	(Lopez-Escamez et al., 2007) [67] (Chiarella, Petrolo and Cassandro, 2015) [9] (Dai et al., 2023) [3]	The association between HLA-DRB1*1101 and MD is highly controversial. This discrepancy may be explained by ethnic and population differences in HLA allele frequencies, as well as the complex structure of the MHC region and interactions with other candidate genes. At present, there is no clear conclusion regarding the role of HLA-DRB1*1101 in the pathogenesis of MD. Its relationship remains an open research question, and clarification will require studies on large, ethnically stratified cohorts, with independent replications and the integration of HLA haplotype analyses [3,9].
		Mediterranean popolation	Increased susceptibility to bilateral MD		
	HLA-Cw*04	Caucasian population	Increased susceptibility to MD	(Khorsandi et al., 2011) [68] (Chiarella, Petrolo and Cassandro, 2015) [9] (Dai et al., 2023) [3]	HLA-Cw*04 cannot be considered a universal causal gene for MD, but rather a conditional susceptibility factor whose contribution becomes relevant only within specific genetic and ethnic backgrounds. This population variability highlights the need to interpret HLA findings through ethnically stratified analyses and further reinforces the concept of MD as a multifactorial disorder, in which interactions between immune-related genes and environmental factors play a determining role [64,68].
		Asian population		(López-Escámez et al., 2002) [64] (Dai et al., 2023) [3]	
		Asian population	Not correlated with MD	(Koyama et al., 1993) [66] (Chiarela, Petrolo and Cassandro, 2015) [9]	
	HLA-Cw*07	Italian population	Increased susceptibility to MD	(Melchiorri et al., 2002) [65] (Chiarela, Petrolo and Cassandro, 2015) [9] (Dai et al., 2023) [3]	The presence of HLA-Cw07 may promote the presentation of autoantigens or viral antigens that mimic inner ear structures, triggering cross-reactive immune responses and persistent inflammation. Moreover, the interaction of HLA-Cw07 with other genetic factors or with environmental influences (such as viral infections, allergic conditions, or systemic inflammatory stress) may further increase individual susceptibility and help explain the clinical variability of the disease. HLA-Cw07 is therefore considered a high-risk allele for MD not through a single direct effect, but by modulating the local immune response and promoting an inflammatory milieu that disrupts inner ear homeostasis and contributes to disease development and progression [3,52].
		British population		(Xenellis et al., 1986) [63] (Dai et al., 2023) [3]	
		Spanish population	Non-significant association with MD	(López-Escámez et al., 2002) [64]	
		European population and Japanese population	Uncertain association with MD	(Koyama et al., 1993) [66] (Arweiler, Jahnke and Grosse-Wilde, 1995) [34] (Fung et al., 2002) [116]	
	HLA	Caucasian population	Not correlated with MD	(López-Escámez et al., 2002) [64] (Chiarela, Petrolo and Cassandro, 2015) [9] (Dai et al., 2023) [3]	
	HLA-DQB1	Mediterranean, Spanish population	Not correlated with bilateral MD	(Lopez-Escamez et al., 2007) [67] (Dai et al., 2023) [3]	
	HLA-A*11	Chinese population	Increased susceptibility to MD	(Chan et al., 2018) [117] (Dai et al., 2023) [3]	HLA-A11 has been investigated as a possible immunogenetic factor involved in MD, particularly in Asian populations. This class I HLA allele is involved in the presentation of endogenous antigens to cytotoxic T lymphocytes and may influence susceptibility to aberrant immune responses within the inner ear. However, this association with MD has not been replicated in European populations [3].
	HLA-DRB1*1201	South Korean population	Partially susceptibility to MD	(Koo et al., 2003) [69] (Dai et al., 2023) [3]	In the subgroup of patients without anti–type II collagen antibodies, the frequency of the HLA-DRB11201 allele was significantly higher compared to the control group, suggesting that this allele may confer increased genetic susceptibility to MD independent of collagen autoimmunity. HLA-DRB11201 may be involved in predisposition to MD by influencing how the immune system recognizes and responds to inner ear antigens, supporting an immunogenetic component in the disease’s pathogenesis [9].
	HLA-DRB1*0405	South Korean population	Partially susceptibility to MD	(Koo et al., 2003) [69] (Dai et al., 2023) [3]	HLA-DRB1 analysis in Korean patients with MD showed that HLA-DRB10405 is associated with disease forms accompanied by anti–type II collagen antibodies, suggesting a role for this allele in susceptibility to MD with an autoimmune component. In contrast, HLA-DRB115 did not show a significant association with the disease in the same cohort, indicating that this allele does not directly contribute to predisposition to MD in the studied population. Thus, DRB10405 may represent a genetic risk factor specific to autoimmune forms, whereas DRB115 appears neutral in this context [69].
	HLA-DRB1*15	South Korean population	Correlated with MD	(Yeo et al., 2002) [70] (Dai et al., 2023) [3]	
	HLA-Cw*0303	South Korean population	Partially susceptibility to MD	(Yeo et al., 2002) [70] (Dai et al., 2023) [3]	In a Korean cohort, an increased frequency of HLA-Cw0303 was observed in patients with MD compared to healthy controls, suggesting a possible role of this allele in immunogenetic susceptibility to MD. However, these results come from a relatively small study and have not been consistently replicated across all ethnic groups, indicating that the true role of HLA-Cw0303 in MD remains inconclusive and dependent on the genetic context of the population studied [3].
	HLA-DRB1*1602	Asian population	Increased susceptibility to MD	(López-Escámez et al., 2002) [64] (Dai et al., 2023) [3]	HLA-DRB11602 has been identified as significantly more frequent in Japanese patients compared to other populations. This observation supports the hypothesis that certain class II HLA variants, including DRB11602, may influence how the immune system presents antigens and responds to self-antigens of the inner ear, thereby facilitating immunopathological mechanisms involved in the etiology of MD. However, this association has not been consistently observed across all studied populations, suggesting an influence that depends on the ethnic and genetic context of the cohorts analyzed [64].
			Non-significant association with MD	(Koyama et al., 1993) [66] (Chiarela, Petrolo and Cassandro, 2015) [9]	
	HLA-B27	American population	Increased susceptibility to bilateral MD	(Rawal et al., 2010) [118] (Dai et al., 2023) [3]	HLA-B27, a classical allele associated with spondyloarthropathies and other systemic inflammatory diseases, has been investigated in MD due to its potential to promote aberrant immune responses and persistent inflammation. The presence of HLA-B27 may contribute to the activation of cytotoxic T lymphocytes or the production of proinflammatory cytokines, facilitating damage to the endolymphatic sac epithelium and disrupting endolymphatic homeostasis. Although a direct association with MD has not been consistently demonstrated, this allele remains relevant as an immunogenetic susceptibility marker in an inflammatory phenotype of the disease [3,118].
Aquaporin genes	AQP2	American population	Not correlated with MD	(Mhatre et al., 2002) [77] (Chiarela, Petrolo and Cassandro, 2015) [9]	The functional expression of AQP2, AQP3, AQP4, and AQP5 allows rapid water movement between intracellular and extracellular compartments, preventing excessive endolymph accumulation and the development of EH. Dysregulation or variations in the expression of these channels can disrupt inner ear fluid homeostasis, contributing to the characteristic symptoms of MD. Genetic studies on these genes have yielded mixed results. Some research has reported associations between certain AQP polymorphisms and susceptibility to MD in specific populations, suggesting that genetic variants may influence endolymph transport efficiency and disease predisposition. However, other studies have failed to confirm these correlations in different populations, indicating that the effects of these genes may depend on population-specific genetic backgrounds, interactions with other candidate genes, or environmental factors such as diet and oxidative stress exposure [12,81].
		Asian population		(Maekawa et al., 2010) [75] (Chiarela, Petrolo and Cassandro, 2015) [9]	
		Caucasian population		(Candreia, Schmuziger and Gürtler, 2010) [78]	
	AQP2 rs426496	Brazilian population	Partially correlated with MD	(Lopes et al., 2016) [12] (Dai et al., 2023) [3]	
	AQP3	Caucasian population	Not correlated with MD	(Candreia, Schmuziger and Gürtler, 2010) [78] (Chiarela, Petrolo and Cassandro, 2015) [9]	
	AQP3 rs591810	Brazilian population	Partiallly correlated with MD	(Lopes et al., 2016) [12] (Dai et al., 2023) [3]	
	AQP3 Homozygous c.105G->C	Swiss population	Partially correlated with MD	Candreia, Schmuziger and Gürtler, 2010) [78] (Dai et al., 2023) [3]	
	AQP4 rs2075575	Japanese population	Not correlated with MD	(Nishio et al., 2013) [80] (Dai et al., 2023) [3]	
	AQP5 The variant G allele of rs3736309	Japanese population	Correlated with MD	(Nishio et al., 2013) [80] (Dai et al., 2023) [3]	
	AQP5–1364 A/C	Caucasian population	Not correlated with MD	(Arweiler-Harbeck et al., 2012) [79] (Dai et al., 2023) [3]	
Potassium channel genes	KCNE1 rs1805127rs1805128rs17173510	Brazilian population	Increased susceptibility to MD	(Lopes et al., 2016) [12] (Dai et al., 2023) [3]	Discrepancies between studies may reflect ethnic differences in allele frequencies, phenotypic heterogeneity of the disease, small sample sizes, and insufficient separation of FMD versus SMD forms in some cohorts. Therefore, although the physiological role of KCNE1 and KCNE3 in ion regulation and endolymph homeostasis is clearly biologically plausible, current genetic evidence remains inconsistent. Overall, KCNE1 and KCNE3 remain candidate genes of interest for MD, and future research, ideally multicenter and conducted on large, heterogeneous cohorts, may clarify how these variants influence disease susceptibility and severity [9].
	KCNE1 rs1805127/ 112G/A	Japanese population	Correlated with MD	(Doi et al., 2005) [10] (Chiarela, Petrolo and Cassandro, 2015) [9] (Dai et al., 2023) [3]	
		Caucasian population/ Asiatic population	Not correlated with MD	(Vrabec et al., 2008) [86] (Campbell et al., 2010) [83] (Chiarela, Petrolo and Cassandro, 2015) [9] (Li, Jin and Xu, 2016) [85] (Dai et al., 2023) [3]	
		Finnish population	Correlated with SMD Not correlated with FMD	(Hietikko et al., 2012) [84] (Chiarela, Petrolo and Cassandro, 2015) [9] (Dai et al., 2023) [3]	
	KCNE1 653 C/T	Chinese population	Correlated with SMD	(Dai, Wang and Zheng, 2019) [13] (Dai et al., 2023) [3]	
	KCNE3 492 A/C	Chinese population	Correlated with FMD	(Dai, Wang and Zheng, 2019) [13] (Dai et al., 2023) [3]	
	KCNE3 198T/C/ (rs2270676)	Japanese population	Correlated with MD	(Doi et al., 2005) [10] (Chiarela, Petrolo and Cassandro, 2015) [9] (Dai et al., 2023) [3]	
		Caucasian population	Not correlated with MD	(Campbell et al., 2010) [83] (Chiarela, Petrolo and Cassandro, 2015) [9] (Li, Jin and Xu, 2016) [85] (Dai et al., 2023) [3]	
Other ion transport associated genes	ADD1 rs4961	Italian population	Correlated with MD	(Teggi et al., 2008) [72] (Chiarela, Petrolo and Cassandro, 2015) [9] (Dai et al., 2023) [3]	Disruption of the function of any adducin can lead to excessive endolymph accumulation, electrolyte instability, and increased pressure in the inner ear, resulting in classic symptoms: vertigo, fluctuating hearing loss, and tinnitus. Genetic studies have identified polymorphisms in ADD1 as potential risk factors for MD. Although evidence remains limited and replication across different populations is rare, these genes may help explain the phenotypic heterogeneity and variable treatment responses observed among patients. Even though no association with MD has been identified so far for ADD2 and ADD3, this hypothesis is not universally valid, as these two genes may be part of a complex genetic framework that contributes to disease development. Replication in different populations and multicenter studies are required to draw robust conclusions [3,9].
	ADD2 rs4984	Italian population	Not correlated with MD	(Teggi et al., 2008) [72] (Dai et al., 2023) [3]	
	ADD3 rs3731566	Italian population	Not correlated with MD	(Teggi et al., 2008) [72] (Dai et al., 2023) [3]	
	SIK1 rs3746951	Caucasian population	Correlated with MD	(Teggi et al., 2017) [73] (Dai et al., 2023) [3]	SIK1 is a serine/threonine kinase belonging to the salt-inducible kinase family, involved in regulating active ion transport, particularly via the Na^+^/K^+^-ATPase, as well as in cellular signaling related to metabolism and stress responses. Genetically, SIK1 has been included in candidate gene panels for familial MD, suggesting it may represent a susceptibility locus for the familial form of the disorder. Its role remains a biological hypothesis that requires confirmation through further functional and clinical studies [73].
	ATQ1	Australian population	Not correlated with MD	(Lynch et al., 2002) [88] (Chiarela, Petrolo and Cassandro, 2015) [9]	ATQ1 is a protein involved in regulating cellular morphogenesis, organizing the cytoplasm, and maintaining cytoskeletal structure in various epithelial and neuronal cell types. It contributes to the stabilization of actin filaments and modulates the intracellular transport of proteins and ions, processes essential for the proper functioning of inner ear cells. Although current evidence is limited, ATQ1 remains a gene of interest in genetic studies of MD, especially in combination with other candidate genes involved in ion transport and fluid regulation in the inner ear [9].
Genes encoding immune, inflammatory, and oxidative stress–related proteins	NFKB1 rs3774937	Spanish population	Correlated with the progression of hearing loss in unilateral MD	(Cabrera et al., 2014) [16] (Dai et al., 2023) [3]	The NF-κB signaling pathway plays a central role in regulating inflammatory responses and is a key mediator of both innate and adaptive immune mechanisms. It controls the expression of numerous genes involved in immune activation, cytokine production, cell survival, and stress responses, thereby contributing to immune homeostasis and inflammatory regulation [3].
	NFKB1 rs4648011				
	PTPN22 1858 C/T	Spanish population	Susceptible for MD	(Lopez-Escamez et al., 2010) [94] (Chiarela, Petrolo and Cassandro, 2015) [9] (Dai et al., 2023) [3]	Encodes a lymphocyte-specific tyrosine phosphatase that plays a critical role in immune regulation by exerting a strong inhibitory effect on T-cell receptor signaling. Through this negative regulatory function, PTPN22 contributes to the control of T-cell activation, maintenance of immune tolerance, and prevention of excessive or dysregulated immune responses [94].
	CTLA4 49 A/G	Spanish population	Not correlated with bilateral MD	(Lopez-Escamez et al., 2010) [94] (Dai et al., 2023) [3]	Mediates the inhibition of T-cell activation by interacting with its ligands, CD80 and CD86, on antigen-presenting cells, thereby attenuating costimulatory signaling, limiting T-cell proliferation, and promoting immune tolerance [94].
	TNF-α (rs1800629)	Spanish population American population	Not correlated with MD	(Gázquez et al., 2013) [96] (Chiarela, Petrolo and Cassandro, 2015) [9] (Dai et al., 2023) [3]	TNF-α is a pro-inflammatory cytokine that plays a central role in immune regulation and inflammatory responses. It is involved in the activation of immune cells, promotion of cytokine cascades, and regulation of apoptosis and cell survival, contributing to both innate and adaptive immune processes [96].
	IFN-γ (rs2234688)	Spanish population American population	Not correlated with MD	(Gázquez et al., 2013) [96] (Chiarela, Petrolo and Cassandro, 2015) [9] (Dai et al., 2023) [3]	IFN-γ is a key pro-inflammatory cytokine involved in immune defense and immune regulation. It activates macrophages, enhances antigen presentation, and promotes Th1-type immune responses, playing a crucial role in coordinating innate and adaptive immunity [96].
	MIF (rs35688089)	Spanish population	Partially correlated with MD	(Gázquez et al., 2013) [96] (Chiarela, Petrolo and Cassandro, 2015) [9] (Dai et al., 2023) [3]	MIF is a pro-inflammatory cytokine that regulates innate and adaptive immune responses. It promotes immune cell activation, sustains inflammatory signaling, and counteracts the immunosuppressive effects of glucocorticoids, thereby contributing to the persistence of inflammation [96].
		American population	Not correlated with MD		
	MIF-173	Caucasian population	Correlated with MD	(Yazdani et al., 2013) [97] (Chiarela, Petrolo and Cassandro, 2015) [9]	Pro-inflammatory factor
		Japanese population	Susceptible for MD	(Arweiler-Harbeck et al., 2012) [79] (Dai et al., 2023) [3]	
	TLR10 (rs11096955)	Spanish and Italian population	Protective effect for MD	(Requena et al., 2013) [15] (Dai et al., 2023) [3]	TLR10 is a member of the Toll-like receptor family, a class of key protein molecules involved in innate (nonspecific) immunity. It plays a critical role in recognizing pathogen-associated molecular patterns (PAMPs), initiating immune signaling cascades, and modulating inflammatory responses. By contributing to the early detection of pathogens and the activation of immune defenses, TLR10 helps orchestrate both the magnitude and the balance of innate immune responses [15].
	CD32A (rs1801274)	Mediterranean and Spanish population	Not correlated with MD	(Lopez-Escamez et al., 2011) [119] (Chiarela, Petrolo and Cassandro, 2015) [9] (Dai et al., 2023) [3]	CD16 and CD32 are transmembrane glycoproteins that function as low-affinity Fc receptors for immunoglobulins, playing critical roles in immune regulation. These receptors are expressed on various immune cells, including natural killer cells, macrophages, and neutrophils, where they mediate antibody-dependent cellular cytotoxicity, phagocytosis, and the clearance of immune complexes. By linking the humoral and cellular arms of the immune system, CD16 and CD32 contribute to the activation, modulation, and fine-tuning of immune responses, as well as to the regulation of inflammation [119].
	CD16A (rs396991)				
	NOS (rs41279104)	Mediterranean and American population	Not correlated with MD	(Gazquez et al., 2011) [103] (Chiarela, Petrolo and Cassandro, 2015) [9] (Dai et al., 2023) [3]	NOS1 is an enzyme primarily expressed in neuronal tissues that produces nitric oxide (NO), a signaling molecule involved in neurotransmission, vasodilation, and the regulation of blood flow. NOS1 also plays a role in modulating immune responses and cellular signaling under physiological and stress conditions [3,9]. NOS2 is an enzyme expressed in immune and other cell types in response to inflammatory stimuli. It generates large amounts of nitric oxide, which functions as a potent antimicrobial and immunoregulatory molecule, contributing to pathogen defense, inflammation, and immune signaling [3].
	NOS2A (rs3833912)				
	IL1A rs1800587	Japanese population	Susceptible for MD	(Furuta et al., 2011) [95] (Dai et al., 2023) [3]	IL-1α and IL-1β are key pro-inflammatory cytokines that play a central role in orchestrating the immune response. They act as signaling molecules to transmit information between cells, activate and regulate the function of immune cells, and promote the activation, proliferation, and differentiation of T and B lymphocytes. By driving inflammatory processes and coordinating both innate and adaptive immunity, IL-1α and IL-1β are critical mediators of host defense, immune regulation, and tissue homeostasis [95,102].
	IL1B rs16944		Not correlated with MD		
	HSPA1A 190 G/C	Japanese population	Susceptible for MD	(Kawaguchi, Hagiwara and Suzuki, 2008) [98] (Chiarela, Petrolo and Cassandro, 2015) [9] (Dai et al., 2023) [3]	HSPA1A encodes a member of the heat shock protein 70 (HSP70) family, which are intracellular protective proteins produced by the body in response to various forms of cellular stress, such as heat, oxidative stress, and inflammation. HSPA1A plays a crucial role in protein folding, preventing aggregation of damaged proteins, and assisting in the refolding or degradation of misfolded proteins. In addition, it helps maintain cellular homeostasis, supports cell survival under stress conditions, and modulates immune responses, making it a key component of the cellular stress response and protective mechanisms [9,13,101].
	GPX1 (rs1050450)	Japanese population	Not correlated with MD	(Teranishi et al., 2012) [120] (Dai et al., 2023) [3]	GPX1 plays a crucial role in maintaining redox balance, preventing cellular injury, and modulating oxidative stress–related signaling pathways, thereby supporting cell survival and overall tissue health [3]. PON1 hydrolyzes lipid peroxides and toxic organophosphates, protecting cells and tissues from oxidative damage and inflammation. PON1 contributes to cardiovascular health and helps maintain the integrity of lipoproteins in the bloodstream [121]. PON2 protects cells from oxidative stress by reducing reactive oxygen species (ROS) and lipid peroxidation, thereby supporting cellular homeostasis and modulating inflammatory and metabolic responses. Unlike PON1, PON2 functions primarily within cells rather than in circulation [122]. SOD2 is essential for maintaining mitochondrial function, preventing oxidative damage to cellular components, and supporting energy metabolism and cell survival under stress conditions [123].
	PON1 (rs662, rs854560)				
	PON2 (rs7493)				
	SOD2 (rs4880)				
	HRH4 rs77485247	Chinese population	Associated with vertigo in MD and pro-inflammatory factor levels in blood	(Qin et al., 2019) [92] (Dai et al., 2023) [3]	HRH4 is a G-protein–coupled receptor that is highly expressed in cells of the immune system, including mast cells, eosinophils, and dendritic cells. It plays a key role in mediating immune and inflammatory responses by regulating the production and release of pro-inflammatory cytokines and chemokines. HRH4 contributes to the recruitment and activation of immune cells at sites of inflammation, modulates hypersensitivity reactions, and is involved in the pathophysiology of allergic and autoimmune diseases [3].
	RANTES–403 A	Iranian population	Protective for male with MD	(Yazdani et al., 2015) [93] (Dai et al., 2023) [3]	Associated with inflammatory disease
	TNF-α–238 A/G	Iranian population	Susceptible for MD	(Kouhi et al., 2021) [124] (Dai et al., 2023) [3]	Pro-inflammatory factor gene
	PARP-1	Spanish population	Not correlated with MD	(Lopez-Escamez et al., 2009) [125] (Chiarela, Petrolo and Cassandro, 2015) [9]	PARP-1 is a nuclear enzyme essential for maintaining genomic integrity, involved in DNA repair, transcriptional regulation, and modulation of cellular responses to oxidative stress. In cochlear and vestibular cells, PARP-1 detects DNA damage and activates repair pathways that influence both cell survival and local inflammatory responses. In the context of MD, it has been hypothesized that PARP-1 activity could contribute to cellular degeneration of the cochlear and vestibular epithelium, particularly under conditions of oxidative stress or chronic inflammation. Although these mechanisms provide a plausible biological basis for PARP-1 involvement, no direct correlation between PARP-1 variants and MD has been demonstrated to date [125].
Other genes and chromosomes	12p12 PIK3C2G	Caucasian population	Susceptible for MD	(Klar et al., 2006) [54] (Chiarela, Petrolo and Cassandro, 2015) [9]	PIK3C2G gene, located on the short arm of chromosome 12 (12p12), encodes a class II phosphatidylinositol 3-kinase (PI3K-C2γ) involved in phosphoinositide-dependent intracellular signaling. These signaling pathways play an important role in regulating vesicular transport, membrane permeability, cellular metabolism, and cellular stress responses. In the context of the inner ear, PI3K signaling pathways are considered essential for maintaining ionic homeostasis and endolymphatic fluid volume, processes that are fundamental for the normal function of the cochlea and the vestibular system. Dysregulation of these mechanisms may contribute to the development of EH. PIK3C2G is currently regarded as a candidate gene with plausible biological relevance; however, further functional and genetic studies are required to clarify its precise contribution to the development and progression of MD [54].
	12p12.3	Caucasian population	Not correlated with MD	(Hietikko et al., 2011) [126] (Chiarela, Petrolo and Cassandro, 2015) [9]	12p12.3 region is located on the short arm of chromosome 12 and contains several genes involved in essential biological processes, such as intracellular signaling, regulation of inflammatory responses, epithelial function, and control of ionic homeostasis. These mechanisms are of major interest in the context of inner ear pathophysiology. Moreover, genetic variations within the 12p12.3 region may modulate the response to environmental factors, including inflammation, oxidative stress, or hormonal imbalances, thereby contributing to the multifactorial and heterogeneous nature of MD [126].
	Chromosome 5	Caucasian population	Correlated with MD	(Arweiler-Harbeck et al., 2011) [18] (Chiarela, Petrolo and Cassandro, 2015) [9]	Chromosome 5 contains a significant number of genes involved in biological processes relevant to inner ear function, including the development and maintenance of sensory structures, regulation of ionic homeostasis, immune responses, and control of inflammation. For this reason, chromosome 5 has been investigated in several genetic studies as a potential contributor to susceptibility to MD, particularly in familial forms or cases with bilateral involvement. Additionally, chromosome 5 harbors genes involved in both innate and adaptive immune responses, supporting the hypothesis of an inflammatory or autoimmune component in the pathophysiology of MD [9].
	HCFC1	Caucasian population	Not correlated with MD	(Vrabec et al., 2008) [86] (Chiarela, Petrolo and Cassandro, 2015) [9]	In the context of MD, HCFC1 has been proposed as a candidate gene due to its role in immune responses and the potential involvement of viral infections in disease pathogenesis. Genetic studies have shown that certain HCFC1 SNP variants are more frequent in patients with MD, suggesting that they may increase susceptibility by affecting the function of the endolymphatic sac or by modulating the local immune response. However, there is no definitive evidence supporting a clear association with MD, and larger studies are needed to replicate these findings [86].
Genes associated with autoimmunity and autoinflammation	MEFV	Chinese population	Genes with uncertain significance for MD	(Zou et al., 2023) [91]	Given that MEFV mutations are well known as markers of autoinflammatory diseases and have also been described in certain autoimmune pathologies, it is plausible that the inflammatory dysregulation associated with these variants may also influence the course of MD [91].
	COL7A1	Chinese population	Genes with uncertain significance for MD	(Zou et al., 2023) [91]	This gene may be involved in the presence of a comorbidity in which MD coexists with hearing impairment within the auditory neuropathy spectrum, reflecting the complexity and heterogeneity of the genetic mechanisms involved in an autoimmune background [91].
	COLEC11	Chinese population	Genes with uncertain significance for MD	(Zou et al., 2023) [91]	The impact of mutations on immune recognition mechanisms may indirectly contribute to the inflammatory susceptibility of the inner ear [91].
	ADA	Chinese population	Genes with uncertain significance for MD	(Zou et al., 2023) [91]	ADA deficiency has also been associated with sensorineural hearing loss [91].
	RAG2	Chinese population	Genes with uncertain significance for MD	(Zou et al., 2023) [91]	The presence of such variants in patients with MD may contribute to a vulnerable immunological background that favors the development of chronic inflammation [91].
	RNF31	Chinese population	Genes with uncertain significance for MD	(Zou et al., 2023) [91]	Variants of this gene have been linked to immunodeficiency and autoinflammation [91].
	FAT4	Chinese population	Genes with uncertain significance for MD	(Zou et al., 2023) [91]	At the molecular level, FAT4 is involved in Hippo signaling and cell–cell interactions that can modulate immune cell infiltration and activation. In the context of MD, a functional alteration of FAT4 could disrupt these immune and vascular regulatory mechanisms, promoting a pro-inflammatory environment or microvascular dysfunction in the inner ear, factors that may contribute to the development of EH [91].
	RAB27A	Chinese population	Genes with uncertain significance for MD	(Zou et al., 2023) [91]	It regulates the transport and exocytosis of secretory granules in immune cells, including neutrophils and eosinophils, influencing the release of proinflammatory mediators that can modulate both local and systemic immune responses. Dysregulation of these processes could contribute to a persistent inflammatory response or disproportionate immune activation in the context of cellular stress in the inner ear, facilitating micro-inflammation or autoinflammation associated with EH [91].
	FANC	Chinese population	Genes with uncertain significance for MD	(Zou et al., 2023) [91]	Proteins encoded by FANC genes are part of a complex that responds to DNA damage and oxidative stress, and disruptions in these processes can create a proinflammatory environment, increase reactive oxygen species, and trigger abnormal immune activation—conditions capable of impairing the blood-labyrinth barrier or enhancing local inflammation in the inner ear. In this context, FANC variants may contribute to susceptibility to MD not through a classic DNA repair defect, but by facilitating exaggerated systemic or local inflammation when the inner ear is exposed to stress, indirectly influencing the development of EH [91].
	TLR3	Chinese population	Genes with uncertain significance for MD	(Zou et al., 2023) [91]	TLR3 encodes Toll-like receptor 3, which recognizes double-stranded nucleic acid molecules and activates proinflammatory signaling pathways via NF-κB and type I interferons, promoting the production of cytokines and chemokines. In the inner ear or in resident immune cells, a functional variant of TLR3 could alter the response to DAMPs (damage-associated molecular patterns) or viral stimuli, initiating or amplifying local inflammation. This innate immune activation may contribute to dysfunction of tissue barriers [91].
	LPIN2	Chinese population	Genes with uncertain significance for MD	(Zou et al., 2023) [91]	Encodes lipin-2, a lipid phosphatase involved in regulating lipid metabolism, cellular stress responses, and the control of inflammatory pathways, including modulation of cytokine signaling and inflammasome activation. Functional alterations in LPIN2 can disrupt the balance between metabolic processes and proinflammatory responses, creating a systemic or local environment prone to persistent inflammation. In the context of the inner ear, such dysfunctions may amplify microvascular inflammation and compromise the blood-labyrinth barrier, promoting the development of EH [91].
	NBAS	Chinese population	Genes with uncertain significance for MD	(Zou et al., 2023) [91]	These disorders have been associated with cellular stress and the amplification of inflammatory processes at the level of the endoplasmic reticulum [91].

MICA—MHC class I chain-related gene A; NK—Natural Killer cells; EH—Endolymphatic hydrops; HLA—Human Leukocyte Antigen; MHC—Major histocompatibility complex; AQP—Aquaporine; KCNE—Potassium Voltage-Gated Channel Subfamily E; ADD—Adducin; SIK1—Salt-Inducible Kinase 1; ATQ—Antiquin; NFKB1—Nuclear Factor Kappa B Subunit 1; PTPN22—Protein Tyrosine Phosphatase Non-Receptor Type 22; CTLA4—Cytotoxic T-lymphocyte-associated protein 4; CD80—Cluster of Differentiation 80; CD86—Cluster of Differentiation 86; TNF-α—Tumor necrosis factor; IFN-γ—Interferon-gamma; MIF—Macrophage Migration Inhibitory Factor; TLR10—Toll-like receptor 10; CD32A—Cluster of Differentiation 32A; CD16A—Cluster of Differentiation 16A; NOS—Nitric Oxide Synthase; NO—Nitric oxide; IL1A—Interleukin-1 alpha; IL1B—Interleukin-1 beta; HSPA1A—Heat Shock Protein Family A Member 1A; GPX1—Glutathione peroxidase-1; PON—Paraoxonase; ROS—Reactive oxygen species; SOD—Superoxide dismutase; HRH4—Histamine Receptor H4; RANTES—Regulated upon Activation, Normal T cell Expressed and Secreted; PARP-1—Poly (ADP-ribose) polymerase 1; HCFC1—Host Cell Factor C1; SNP—Single-nucleotide polymorphism; MEFV—Mediterranean FeVer; COL7A1—Collagen, type VII, alpha 1; COLEC11—Collectin Subfamily Member 11; ADA—Adenosine deaminase; RAG2—Recombination Activating Gene 2; RNF31—RING finger protein 31; FAT4—FAT atypical cadherin 4; RAB27A—RAB27A, Member RAS Oncogene Family; FANC—Fanconi anemia complementation group; TLR3—Toll-like receptor 3; LPIN2—Lipin-2; NBAS—Neuroblastoma Amplified Sequence.

## 3. Limitations, Discussion, and Conclusions

The aim of our article was to highlight the main genetic factors discussed over time and to open new perspectives for future studies that could help clarify research directions. In the future, we intend to conduct a meta-analysis to closely examine the inconsistencies among existing studies and to identify specific explanations, as well as potential solutions. The role of genes in MD appears to be a fascinating field; however, it is imperative to identify the key genes on which genetic screening should focus, a task complicated by contradictory findings and divergent opinions across studies.

All candidate genes analyzed in scientific literature can reasonably be linked to the pathophysiology of MD, and each published study proposes a plausible mechanism by which these genes might influence disease progression. Nevertheless, the validity of these associations critically depends on their confirmation in subsequent investigations conducted in independent cohorts. A major challenge in evaluating these results lies in the fact that, in most studies, patients with familial and sporadic forms of the disease were included in the same analyses, without a clear distinction between these etiologically distinct entities [9].

Can we conclude that a gene is a true risk factor? No, not if the evidence is weak or contradictory. A single significant association in a small study is not sufficient to confirm that a gene is a true risk factor. However, this can be established if there are consistent replications across multiple independent studies, if meta-analyses are performed that combine data from different studies to increase statistical power, and if functional studies demonstrate clear biological mechanisms through which the gene influences MD. For a gene to be considered a true risk factor, more than a simple statistical association is required [127].

One problem identified in these studies relates to the small sample sizes, which lead to a higher likelihood of false-positive or false-negative results. Frequently, variants with strong effects can be detected in small samples, whereas variants with modest effects require very large cohorts to achieve adequate statistical power [7]. Some studies suggest a significant association with a specific gene, indicating that the effect may be more pronounced in certain subgroups. Each study may include a population with distinct genetic, ethnic, and clinical characteristics, which can influence the results. For example, an Asian population may have a different genetic profile compared to a European population, and these differences can affect susceptibility to MD.

Another problematic aspect is the lack of replication of previously reported genes, which weakens the consistency of the evidence available to date. Studies conducted in different populations, across diverse ethnic backgrounds and age groups, often yield contradictory results. Without interpopulation validation, the generalizability of findings remains limited. Moreover, the tendency to focus on individual genes represents a reductionist and potentially biased approach that overlooks the complexity of genetic interactions. A more comprehensive and hypothesis-free alternative would be genome-wide association studies (GWAS), which are better suited to capture the polygenic nature of susceptibility. Methodological errors also need to be systematically addressed in all studies to ensure the consistency and reliability of results [8].

While correlations between mutations and phenotypes have already been established for other genetic diseases, in the case of MD, the use of modern exome and whole-genome sequencing technologies (WES/WGS) provides a significant opportunity to improve genetic diagnosis for both familial and sporadic forms, where causal mutations remain unknown. As these technologies are applied to larger cohorts, it is expected that new genes and genetic variants associated with MD will be identified. In MD, where genetic mechanisms are often polygenic and context-dependent, WES is useful for identifying rare variants with major effects, particularly in familial forms. WGS is becoming increasingly relevant for exploring non-coding variants, HLA haplotypes, and regulatory regions involved in the expression of genes associated with inner ear homeostasis [8].

WES analyzes all coding regions of the genome, namely the exons, which represent approximately 1–2% of the human genome. This approach focuses on protein-coding sequences, where most known pathogenic variants are located. WES is effective in identifying mutations with major functional impacts on proteins and is widely used in studies of rare genetic diseases and in clinical research. A limitation of this technique is that it does not detect variants in non-coding regions and has reduced sensitivity for structural variations, repeat sequences, or GC-rich regions.

WGS analyzes the entire genome, including both coding and non-coding regions (introns, regulatory regions, and intergenic regions). This method provides the most comprehensive view of genetic variation and allows the identification of mutations that may affect the regulation of gene expression. The advantages of this technique include the detection of both coding and non-coding variants, identification of complex structural variations, insertions, deletions, and genomic rearrangements, and more uniform coverage of the genome. However, the costs are high, and the interpretation of non-coding variants is often challenging. As the costs of whole-genome sequencing continue to decrease, a paradigm shift in the genetic research of MD is warranted. The integration of comprehensive genomic analyses, combined with detailed clinical and phenotypic data, will allow mapping of the complex genetic architecture of this disease and, ultimately, pave the way toward personalized medicine in the diagnosis and management of patients with MD [8].

In a field involving numerous genes, questions arise regarding which genes should be prioritized in research. Studies with larger sample sizes and adequate statistical power are far more reliable. If a study finds a significant association in a small group of participants, this may be due to chance or a statistical error. In contrast, multicenter studies or studies replicated in another cohort of participants are more credible. A valid and trustworthy study is one that can be replicated by other researchers in different contexts or populations. Replication studies are essential for establishing the validity of a genetic association. It is important to assess whether the method used to identify genetic associations is robust and correctly applied. Studies that employ advanced techniques to correct for multiple comparisons (e.g., false discovery rate—FDR) and control for confounding variables are generally more reliable [3,127].

Studies published in high-impact journals (recognized within the field) and subjected to rigorous peer review are more likely to be credible. When many variables (genes and polymorphisms) are tested simultaneously, as is the case in large-scale association studies or genome-wide association studies (GWAS), there is a higher probability of detecting coincidental associations. This phenomenon is known as a Type I error (false positive). Studies with positive or significant results are published more frequently than those with negative or neutral results. This phenomenon, known as publication bias, can lead to an overestimation of the relevance of a genetic association.

If genes are not true risk factors, genetic testing could become unnecessary. Patients might undergo costly tests without practical benefit, potentially leading to a false sense of security (if the test is negative) or unnecessary anxiety (if the test is positive but clinically insignificant). By focusing exclusively on genetic factors and neglecting other relevant risk variables, clinical practice risks becoming unbalanced and guided by an incomplete or incorrect model of causality. Moreover, if studies propose genotype-based protective therapies and these genes have no real impact, such interventions may fail, clinical trials could be invalidated, and confidence in personalized medicine could diminish. Therefore, rigorous validation, replication of findings, and multifactorial research approaches are essential to ensure both scientific integrity and clinical utility.

Understanding the genetic basis of MD remains limited, and the hereditary mechanisms involved are still insufficiently elucidated. This lack of clarity is also reflected in clinical practice, where many ENT physicians are not fully aware of the importance of collecting family history and reconstructing pedigrees—essential tools for identifying and characterizing familial cases of MD. Genetic testing in MD is still at an early stage, with no single gene or set of genetic markers validated across different populations to date. The identification and interpretation of rare variants in candidate genes represent an important step in the development of the disease’s genetic diagnosis. However, progress in this field is hindered by the lack of large population-specific databases, tailored to different geographic and ethnic regions, which could serve as a reference for evaluating the clinical significance of the variants identified [7,8].

Focusing on the cardinal symptoms of the disease, such as progressive hearing loss and tinnitus, could contribute to defining a standardized framework for genetic diagnosis. However, the development of these criteria requires the involvement of a multidisciplinary team of clinicians and geneticists to establish uniform interpretation protocols in accordance with international guidelines [5,8]. Therapeutic and rehabilitation strategies in MD should include, in addition to vertigo and hearing loss management, integrated programs for fall prevention and tinnitus management to reduce the overall impact of the disease on patient functioning and well-being. The diagnosis of MD remains essentially clinical and variable, based on a combination of fluctuating symptoms and audio vestibular investigations that are not always standardized. This reliance on the clinician’s judgment introduces a considerable risk of misclassification, either through inclusion of similar peripheral vertigo forms or underdiagnosis of cases with atypical or incomplete presentations [5,128].

Recent studies indicate that chronic vestibular disorders, including MD, are frequently associated with psychiatric symptoms such as anxiety, depression, and panic attacks, which are considered both consequences of the disease and factors that may exacerbate the course of vestibular symptoms. Molnár and colleagues showed that patients experiencing vertigo or persistent dizziness report significantly higher depression scores and a considerable reduction in quality of life. The psychological impact of MD is largely determined by the unpredictable and recurrent nature of vertigo episodes. Patients often develop a fear of experiencing a new episode in social or professional settings, a phenomenon described in the literature as “vestibular anticipatory anxiety.” This psychological state may lead to avoidance of daily activities, limitation of mobility, reduced social participation, and decreased personal autonomy. Over time, these restrictions can contribute to the development of depressive symptoms, feelings of loss of control, and reduced self-esteem [129].

Psychiatric symptoms may in turn influence the course of MD, creating a vicious cycle between the vestibular and psychological components. Studies have shown that anxiety and depression can increase the subjective perception of vertigo severity and may reduce the capacity for vestibular compensation. Patients with higher levels of anxiety or depression tend to report a more severe vestibular handicap and a slower clinical recovery. Thus, the psychological component represents not only a consequence of the disease but also a factor that may contribute to the persistence and amplification of symptoms. The consequences for quality of life are therefore profound and multidimensional. Patients with MD frequently experience significant reductions in quality of life across several domains, including mobility, social activities, occupational functioning, and mental health. Limitations in daily activities, uncertainty regarding disease progression, and the psychological burden of symptoms contribute to an overall negative perception of health status and of the ability to maintain a normal lifestyle [129,130].

In this context, the therapeutic approach to MD should extend beyond the control of vestibular and auditory symptoms and include the systematic evaluation of psychological components. The integration of psychological support, cognitive-behavioral therapy, and stress-management strategies may help reduce anxiety and depression and improve patients’ adaptation to the disease. A multidisciplinary approach that combines otological treatment with psychological interventions and patient education may reduce the overall burden of the disease and significantly improve the quality of life of patients with MD [129].

Literature also faces challenges due to the lack of uniformly applied diagnostic criteria. Over time, various organizations, such as the AAO-HNS and the Bárány Society, have proposed different definitions of the disease with varying degrees of specificity. Some criteria require strict documentation of low-frequency hearing loss, while others allow the inclusion of “probable” or “possible” cases, reducing the international comparability of epidemiological studies. Consequently, a case considered confirmed in one region may not meet the criteria applied in another [128].

The most recent guidelines developed by the Bárány Society represent an important step toward standardizing the diagnostic criteria for MD and harmonizing the terminology used in clinical and epidemiological studies. These recommendations were established through an international consensus involving several major scientific organizations, including the Bárány Society, the European Academy of Otology and Neurotology (EAONO), the American Academy of Otolaryngology–Head and Neck Surgery (AAO-HNS), the Japan Society for Equilibrium Research, and the Korean Balance Society. The primary objective of these guidelines was to define clear and reproducible diagnostic criteria for MD, enabling comparability between studies and facilitating the accurate identification of patients included in clinical research and medical practice [131].

A central feature of the guidelines is the introduction of a simplified diagnostic classification that includes two main categories: “definite Ménière’s disease” and “probable Ménière’s disease.” The diagnosis of definite MD requires the presence of at least two spontaneous episodes of vertigo lasting between 20 min and 12 h, associated with audiometrically documented sensorineural hearing loss, predominantly affecting low or mid frequencies, as well as fluctuating aural symptoms such as tinnitus, aural fullness, or changes in hearing in the affected ear. In addition, these manifestations must not be better explained by another vestibular disorder [131].

The category of probable MD was introduced to include patients who present a suggestive clinical picture but do not yet meet all the criteria required for the definite form of the disease. This category includes patients with recurrent episodes of vertigo or dizziness lasting between 20 min and 24 h, associated with fluctuating aural symptoms, in the absence of an alternative diagnosis. The introduction of this classification reflects the recognition that MD may evolve progressively and that, in the early stages, clinical manifestations may be incomplete or difficult to differentiate from other vestibular disorders [131].

The Bárány Society guidelines also emphasize the importance of differential diagnosis in the evaluation of patients with episodic vertigo. Conditions such as vestibular migraine, benign paroxysmal positional vertigo, vestibular neuritis, or other inner ear disorders may present with similar manifestations and must be excluded through appropriate clinical evaluation and investigations. In this context, the use of standardized criteria facilitates the correct identification of MD and reduces the risk of overdiagnosis or misdiagnosis [131].

Overall, the Bárány Society guidelines provide a solid methodological framework for the diagnosis and classification of MD, contributing to the harmonization of diagnostic criteria at an international level. The implementation of these criteria in both research and clinical practice allows a more precise characterization of patients, improves comparability between studies, and supports the development of therapeutic strategies better adapted to the phenotypic diversity of the disease.

A possible step toward the clinical implementation of genetic data in MD is the definition of a preliminary screening panel intended for high-risk patients, particularly those with a positive family history. Based on current evidence, an exploratory panel could include genes associated with familial forms of MD or overlapping phenotypes of hereditary hearing loss, as well as genes involved in maintaining the structural integrity of the inner ear. The inclusion of genes implicated in immune response regulation or endolymphatic permeability may also be justified, given the growing body of evidence supporting the contribution of chronic inflammation to disease pathogenesis.

Such a panel should not be interpreted as a definitive diagnostic tool, but rather as a risk-stratification instrument. In clinical practice, genetic findings could be correlated with phenotypic features (age at onset, bilaterality, severity of hearing loss, frequency of vertigo attacks, and autoimmune comorbidities) to identify biologically distinct patient subgroups. This approach would allow differentiation between predominantly degenerative forms, those with an immune-mediated substrate, and those associated with structural defects of the inner ear, thereby facilitating personalized monitoring and the selection of patients for targeted therapies or clinical trials. In the future, integrating genetic data with immunological biomarkers and imaging parameters may form the basis of a multimodal personalized medicine model in MD. Although the evidence remains emerging, the definition of a preliminary screening panel represents a pragmatic first step toward translating genetic findings into clinical practice and developing management algorithms tailored to the individual biological profile of each patient.

Integration of genetic data with clinical phenotypes in MD can be achieved through a multistep stratification model that combines molecular information with the evolutionary characteristics of the disease. In a first stage, the identified genetic variant can be classified according to the suggested biological mechanism (for example, genes involved in inner ear development, ionic homeostasis, immune response, or neuro-sensory processes). This classification provides an initial level of pathogenic hypothesis.

In a second stage, genetic findings can be correlated with major clinical parameters such as age at onset, unilateral or bilateral involvement, rate of hearing-loss progression, frequency of vertigo attacks, and the presence of autoimmune or migraine comorbidities. For instance, the identification of variants in genes associated with hereditary hearing loss may suggest a phenotype with early onset and progressive auditory decline, whereas variations in immune-related genes may be more frequently associated with fluctuating, bilateral forms or with an autoimmune background.

In a third stage, integrating these data may allow the definition of biological patient subgroups, such as predominantly structural, immune-mediated, or neuro-sensory forms. This stratification could have practical consequences, including tailoring the intensity of audiological monitoring, prioritizing imaging investigations, selecting patients for immunomodulatory therapies, or enrolling them in mechanism-oriented clinical trials.

Although this model remains conceptual and requires prospective validation, it provides a pragmatic framework for translating genetic findings into clinical decision-making and for developing a personalized-medicine approach in MD.

Currently, there is no solid evidence that a single gene or unique genetic marker could be responsible for the onset of the disease. This lack of consistency may have several explanations: many studies are case–control in design, which limits their inferential power; susceptibility genes may vary between different populations; FMD may have genetic characteristics distinct from SMD, yet most research does not separate these two categories; and the etiology of MD is likely polygenic, with complex interactions between genetic and environmental factors [132].

The study by Hietikko et al. provides a comprehensive evaluation of some of the most frequently mentioned genes in relation to MD [84]. The authors tested variants in the AQP2, KCNE1, KCNE3, COCH, HCFC1, and ADD1 genes in a group of 59 patients (38 with sporadic forms and 21 without known familial linkage) and in a cohort of 98 patients. The results showed that only the rs1805127 polymorphism in the KCNE1 gene retained statistical significance after applying strict corrections for multiple testing. For the other genes investigated, no convincing association with MD was identified, and haplotype and diplotype analyses did not reveal statistically relevant patterns. These observations underscore, once again, the need for large, well-controlled genetic studies that are stratified by clinical subgroups to clarify the actual role of these candidate genes in the etiology of MD, as the mechanism underlying EH appears to be influenced by these genes. These genes should be prioritized in studies, and the results obtained should be replicated and analyzed to determine whether they could indeed represent important biomarkers for future disease diagnosis [9,133].

Teggi et al. reported statistical significance for a polymorphism in the adducin-1 (ADD1) gene, rs4961, suggesting that the minor allele could contribute to MD in heterozygous individuals [72]. Interestingly, the same SNP has previously been associated with hypertension, myocardial infarction, and stroke [134]. However, patients with MD do not exhibit an increased risk for these conditions, raising doubts about the relevance of the proposed mechanism for MD. Nevertheless, the existence of an animal model for hypertension associated with ADD1 mutations provides a future opportunity to study effects on hearing and inner ear histology, which is particularly important for genes with statistically significant associations [7].

Some early studies suggested an association between the Cw7 antigen and MD [63], but results were inconclusive in other studies [34,66,116] only two studies replicated this association [64,65], while others reported its absence, which drew attention to chromosome 6p as a candidate region without identifying a specific gene [7]. The HLA-Cw*07 gene should be prioritized in MD studies because it occupies a unique functional position at the intersection of adaptive and innate immunity, a feature particularly relevant to disease pathophysiology. Unlike many other HLA genes, HLA-C is the main ligand for killer immunoglobulin-like receptors (KIRs) expressed on NK cells and certain subpopulations of cytotoxic T lymphocytes.

HLA-Cw*07 alleles play a central role in modulating the immune response, particularly in the context of viral infections, which are a known trigger of MD. HLA-C belongs to the class I HLA molecules and is involved in presenting viral antigens to cytotoxic T cells (CD8+), as well as regulating natural killer (NK) cells through killer immunoglobulin-like receptors (KIRs) [3,8].

Genetic variability at the HLA-Cw07 locus can influence the activation threshold of NK cells and cytotoxic T lymphocytes. This determines how efficiently the body recognizes and eliminates virus-infected cells, as well as how local inflammation is controlled. In carriers of certain HLA-Cw07 variants, the immune response may be more intense or persistent, promoting chronic activation of inflammation within the endolymphatic sac. This local inflammation can disrupt fluid and ion homeostasis, contributing to endolymph accumulation and the occurrence of vertigo episodes characteristic of MD [3,91].

Thus, HLA-Cw*07 not only affects susceptibility to viral infections but also modulates how the immune response is calibrated in the inner ear. This gene–environment interaction highlights how genetic predisposition can determine the intensity and duration of infection-induced inflammatory responses, thereby increasing the risk of endolymphatic hydrops and the clinical manifestations of the disease [91].

Variations in HLA-Cw*07 can therefore directly influence the immune activation threshold and local immunological tolerance, including in the inner ear, where the balance between protection and inflammation is essential. This capacity to modulate innate immune responses distinguishes it from other HLA genes, which have been studied primarily for their role in antigen presentation to helper T cells. Furthermore, HLA-Cw*07 is one of the most common HLA alleles at the population level and has well-documented associations with multiple inflammatory and autoimmune diseases, suggesting a broader role in regulating immune susceptibility. Given these considerations, we believe greater attention should be given to this gene, as it appears to be a key gene in the pathophysiology of MD. Large, multicenter studies with sufficient statistical power could shed light on this context.

Prioritizing in future studies the genes associated with a protective effect in MD, such as HLA-DRB109, HLA-B44, HLA-DRB113, or HLA-DR2, remains important, as these genes may provide insights into immunological, molecular, or homeostatic mechanisms that limit inflammation and protect the inner ear. However, it should be emphasized that protective associations for each allele have generally been reported in a single study, without consistent replication in other cohorts or populations. Therefore, we cannot assert with certainty that these alleles confer a general protective effect; rather, they indicate a possible biological direction that needs to be validated through additional multicenter studies across diverse populations. The study of alleles with protective effects remains valuable, as it can guide research toward compensatory pathways or beneficial immunological mechanisms and may help in developing preventive or therapeutic strategies, even though their true role in the pathophysiology of MD requires further confirmation.

Genetic linkage analysis has limited utility in the study of complex diseases, as these conditions are not determined by a single genetic variant with a major effect, but rather by the accumulation of multiple variants with individually modest impacts, often interacting with environmental factors. Available evidence indicates that MD also has a predominantly polygenic and multifactorial genetic basis, which limits the ability of linkage studies to identify relevant loci [3].

In this context, association studies represent a more appropriate approach, as they compare the frequency of genetic variants between groups of patients with the same phenotype and unaffected individuals, without requiring large families with multiple affected members. For genetic variants with a small effect on disease risk, classic family-based studies become inefficient. Although transmission disequilibrium testing, which involves patient–parent analysis, can reduce certain biases, the application of this method is limited in diseases with predominantly adult onset, such as MD [7]. While association studies are an essential tool in the genetics of complex diseases, they have well-known limitations in identifying new candidate genes. Issues such as imprecise phenotype definition or overinterpretation of SNPs with marginal effects can lead to false conclusions. Validation of results in an independent population is essential before a preliminary association can be considered credible [5,7,135].

Thus, we can conclude that the genetics of MD is extremely complex, potentially involving inflammation, immunity, metabolism, and the regulation of water and ion balance in the inner ear. However, studies are limited by the low incidence of disease, diagnostic challenges, and the lack of large, diverse cohorts. It is possible that a single gene, or even multiple implicated genes, may not be sufficient to cause the disease on their own. Future research across diverse populations using advanced genetic and epigenetic methods is needed to clarify the mechanisms involved, as well as to carefully analyze how environmental factors interact with a predisposing genetic background, considering possible interactions between genes [3,9].

Non-genetic factors have a significant impact on MD, yet measuring and controlling them remains a major challenge. Although these factors are essential for understanding individual risk, they are difficult to standardize and can vary substantially between patients. Furthermore, their interactions with genetic factors can further complicate the identification of a clear and predictable risk for MD. An integrated approach that combines both genetic and non-genetic factors is therefore essential for a more precise and personalized assessment of MD risk.

Available data suggest that MD results from a complex interaction between genetic susceptibility and non-genetic factors, which influence both the onset and progression of symptomatology. Genetic factors, including HLA variants and polymorphisms in genes involved in inflammation, ion homeostasis, and immune response, establish a baseline biological predisposition that can determine the vulnerability of the vestibular apparatus and the inner ear to endolymphatic imbalances. In this context, non-genetic factors—such as diet, hydration, caffeine and alcohol intake, circadian rhythm disturbances, sleep disorders, stress, exposure to toxins, viral infections, and metabolic or autoimmune comorbidities—do not act in isolation but interact with the genetic background, modulating the phenotypic expression of the disease. These factors can amplify or accelerate the development of endolymphatic hydrops through physiological changes in endolymph volume and pressure, local and systemic immune dysfunctions, neuroendocrine and vascular disturbances, or increased sensitivity to exogenous triggers [128,132].

Thus, non-genetic factors can be considered phenotype modulators, influencing the severity, frequency, and course of vertigo attacks, tinnitus, and the sensation of aural fullness. The interaction with the genetic substrate explains why identical environmental exposures produce different effects across individuals and populations, and why responses to non-pharmacological or dietary interventions can be highly variable. From a clinical perspective, this interdependence between genetic and non-genetic factors suggests that the management of MD should be individualized, integrating genetic evaluation, control of environmental factors, and the adoption of strategies to prevent and stabilize endolymphatic homeostasis [32,128].

A multidisciplinary approach—including nutrition, sleep regulation, stress management, hormonal monitoring, and reduction in exposure to exogenous risk factors—can modulate the phenotypic expression of the disease, limiting progression and symptom severity even in the presence of genetic predisposition. Therefore, MD should not be considered purely genetic or purely environmental; rather, it represents a gene–environment phenomenon, in which hereditary susceptibility defines the biological terrain, and non-genetic factors act as triggers or accelerators of pathophysiological mechanisms, including endolymphatic hydrops, ionic dysfunction, local inflammation, and neuroendocrine dysregulation. This conclusion underscores the importance of an integrated approach targeting both the genetic substrate and modifiable factors to optimize the prognosis of patients with MD [128,132].

Finally, the characterization of truly relevant genetic markers requires validation through experimental models that confirm the physiological impact of identified variants. Understanding the biological mechanisms involved in MD could lead to more accurate diagnosis, finer phenotypic stratification, and, ultimately, the development of novel therapeutic targets capable of altering disease progression.

## Figures and Tables

**Table 1:** Gene associated with familial Ménière’s disease with autosomal dominant inheritance.

Gene	Locus	Studied Population	Involved Study	Remarks
PRKCB (Protein Kinase C Beta)	16p12.2—p12.1	Spanish family	(Martín-Sierra et al., 2016) [38] (Gallego-Martinez and Lopez-Escamez, 2020) [8]	In the context of MD, PRKCB has been suggested to influence inner ear fluid homeostasis and the survival of cochlear and vestibular hair cells by modulating signaling pathways that control endolymph permeability and local inflammatory responses. Genetic data indicate that certain PRKCB variants may be associated with susceptibility to MD in some populations; however, the evidence remains limited and inconsistent. Therefore, PRKCB represents a functionally relevant candidate gene, although its exact role in the pathogenesis of MD is still unclear [38,51].
FAM136 (Family with sequence similarity 136, member A)	2p13.3	Spanish family	(Requena et al., 2015) [37] Gallego-Martinez and Lopez-Escamez, 2020) [8]	Rare variants in FAM136 have been associated with increased susceptibility to FMD, suggesting that the encoded protein may play a role in the function of cochlear and vestibular hair cells or in the homeostasis of inner ear fluids. To date, FAM136 is not well characterized functionally, and the exact mechanisms by which it influences disease onset remain unclear. It is hypothesized that its variants may affect cellular signaling or protection against oxidative stress, thereby facilitating the development of EH [8,52].
DTNA (Dytrobrevin alpha)	18q12 1	Spanish family	(Requena et al., 2015) [37] Gallego-Martinez and Lopez-Escamez, 2020) [8]	DTNA encodes a protein involved in the formation and maintenance of structural complexes at the cellular membrane, playing essential roles in anchoring the cytoskeleton to the membrane and maintaining the integrity of epithelial and hair cells. Genetic studies on FMD have identified rare variants in DTNA in multiplex affected families, suggesting a link between structural alterations of the proteins encoded by this gene and disease predisposition [51]. To date, DTNA is considered a candidate gene for FMD, but its association has been documented only in a few families and has not yet been replicated broadly across diverse populations. Thus, its exact role and molecular mechanism remain under investigation, and the gene’s contribution may be modulated by other candidate genes and environmental factors [8].
SEMA3 D (Semaphorin 3D)	7q21.11	Spanish family	(Martín-Sierra et al., 2017) [39] (Gallego-Martinez and Lopez-Escamez, 2020) [8]	SEMA3D is involved in neuronal axon guidance, vestibular system development, and the maintenance of structural integrity in the inner ear. By regulating cell migration and neural connectivity, SEMA3D can influence the development and organization of the membranous labyrinth, which is critical for endolymph regulation. Functional alterations or rare variants in SEMA3D may disrupt these essential processes, suggesting a mechanism by which familial genetic predisposition could contribute to the phenotype of MD. Genetic studies of FMD have identified rare SEMA3D variants that segregate the disease in certain families, indicating a potential influence of this gene on susceptibility. These findings suggest that SEMA3D likely acts as a modulatory risk factor rather than a monogenic determinant, contributing to the phenotypic heterogeneity observed in FMD [8,51].
DPT (Dermatopontin)	1q24.2	Spanish family	(Martín-Sierra et al., 2017) [39] (Gallego-Martinez and Lopez-Escamez, 2020) [8]	In the inner ear, DPT is primarily expressed in the cells of the endolymphatic sac and duct, structures essential for regulating endolymph volume and pressure. Dysfunction of DPT can compromise the structural integrity of these cells and disrupt endolymph homeostasis, promoting the development of EH, but further research is needed to clarify this area. From a genetic perspective, DPT variants have been proposed as susceptibility factors in FMD. While not considered standalone causal genes, alterations in DPT may increase vulnerability to disease development, especially when interacting with other genes involved in inner ear homeostasis or with environmental factors [51,53].
PIK3CG (Phosphatidylin ositol 3-kinase catalytic gamma)	7q22.3	Swedish families	(Klar et al., 2006) [54] (Gallego-Martinez and Lopez-Escamez, 2020) [8]	PIK3CG, which encodes the catalytic p110γ subunit of class I PI3K, plays a central role in G protein–coupled receptor–dependent cellular signaling, influencing processes such as cell survival, leukocyte migration, and inflammatory responses. In the context of the inner ear, these functions suggest a potential mechanism by which a variant or altered expression of PIK3CG could affect endolymph homeostasis and the response to osmotic or local inflammatory stress. However, to date, genetic studies of FMD have not identified PIK3CG as a recurrent susceptibility gene, indicating that any potential involvement is likely modulatory and dependent on genetic and environmental context, rather than a direct causal factor [8].
HMX2 (H6 Family Homeobox 2)		Finnish families	(Skarp et al., 2019) [40] (Gallego-Martinez and Lopez-Escamez, 2020) [8]	HMX2 encodes a transcription factor involved in the embryonic development of the inner ear, particularly in the differentiation of vestibular structures. Dysfunction of this gene can lead to subtle anomalies in vestibular architecture, which do not result in obvious congenital malformations but may create a biological predisposition for imbalances in endolymph homeostasis. The importance of HMX2 in MD pathogenesis is supported by the correlation between its role in vestibular development and the clinical phenotype observed in patients, which is dominated by recurrent vertigo and progressive vestibular dysfunction rather than early severe hearing loss. It is a recently identified gene, and further studies are needed to clarify its role [40,51].
TMEM55B (Transmembrane Protein 55B)		Finnish families	(Skarp et al., 2019) [40] (Gallego-Martinez and Lopez-Escamez, 2020) [8]	TMEM55B is a gene that encodes a transmembrane protein involved in phosphoinositide metabolism and the regulation of endosomal trafficking in cells. Functionally, TMEM55B acts as a specific phosphatase for phosphatidylinositol-4,5-bisphosphate (PIP2), catalyzing its conversion to PI5P, which affects processes such as endocytosis, intracellular trafficking, and cellular signaling. Through these mechanisms, the protein can modulate cellular homeostasis, cytoskeletal organization, and intracellular signaling. In the context of FMD, TMEM55B has been identified in exome sequencing studies as a rare candidate gene, with certain rare genetic variants found in affected family members, suggesting a possible contribution to disease susceptibility. Essentially, TMEM55B may contribute to susceptibility in a complex genetic context, but its role remains potential rather than definitively confirmed, likely representing just one element of the multiallelic genetic inheritance observed in some affected families [40,51].
CYP2B6 (rs138264188) (Cytochrome P450 family 2 subfamily B member 6)	c.200C>T/p.T67M	Finnish families	(Skarp et al., 2022) [32]	Functional dysregulation of CYP2B6 may influence the ability of cells to respond to oxidative stress, which can affect microcirculation and redox homeostasis in the inner ear. On this basis, the authors proposed that the effects of the rs138264188 variant on CYP2B6 function could predispose individuals to MD by amplifying oxidative stress, thereby facilitating cellular dysfunction and the pathophysiology of EH observed in patients with FMD [32].
GUSB (Glucuronidase beta)	c.323C>T/p.P108L	Finnish families	(Skarp et al., 2022) [32]	An alteration in GUSB function could affect the integrity of the extracellular matrix structure and cellular degradation mechanisms within the membranous labyrinth, thereby facilitating disturbances in fluid balance or increased sensitivity to cellular stress. On this basis, the authors have proposed that GUSB variants may contribute to the etiology of FMD through mechanisms involving lysosomal dysfunction and metabolite accumulation, highlighting the potential role of matrix homeostasis and cellular catabolism in disease pathogenesis [32].
SLC6A7 (Solute carrier family 6 member 7)		Finnish families	(Skarp et al., 2022) [32]	SLC6A7 encodes a solute carrier transporter involved in amino acid uptake and the regulation of cellular homeostasis; its dysfunction may influence oxidative and metabolic balance in inner ear cells. On this basis, SLC6A7 is considered a candidate gene for a role in the FMD, through mechanisms related to intracellular metabolism and transport, with potential involvement in cellular stress responses [32].
ASPM (Assembly factor for spindle microtubules)	(c.5207A>G/p.Q1736R)	Finnish families	(Skarp et al., 2022) [32]	Although ASPM is classically known for its role in mitotic spindle organization during neurogenesis, its association with FMD suggests that disruptions of essential cellular mechanisms, such as cell cycle control and proliferative integrity, may indirectly contribute to dysfunction of inner ear structures or increase susceptibility to complex pathological processes [32].
KNTC1 (Kinetochore associated 1)	(c.5242A>C/p.T1748P)	Finnish families	(Skarp et al., 2022) [32]	The association of this gene with FMD suggests that defects in fundamental cellular integrity mechanisms may contribute to tissue dysfunction in the inner ear. This association supports the concept of heterogeneous genetic etiology for FMD [32].

## Data Availability

No new data were created or analyzed in this study.

## Abbreviations

The following abbreviations are used in this manuscript:

AAO-HNS	Academy of Otolaryngology–Head and Neck Surgery
AD	Autosomal dominant
ADA	Adenosine deaminase
ADD	Adducin
ADH/AVP	Vasopressin or antidiuretic hormone
ASPM	Assembly factor for spindle microtubules
AR	Autosomal recessive
ARMC9	Armadillo Repeat Containing 9
AQP	Aquaporine
AQT	Antiquin
BRCA	BReast CAncer genes
CAG	Cytosine-Adenine-Guanine
CLDN14	Claudin-14
CYP2B6	Cytochrome P450 family 2 subfamily B member 6
CYP1A1	Cytochrome P450 1A1
COCH	Cochlin
COL7A1	Collagen, type VII, alpha 1
COLEC11	Collectin Subfamily Member 11
CTLA4	Cytotoxic T-lymphocyte-associated protein 4
DFNA9	Autosomal dominant non-syndromic hearing loss type 9
DTNA	Dystrobrevin Alpha
DPT	Dermatopontin
EAONO	European Academy of Otology and Neurotology
EH	Endolymphatic hydrops
ENG	Electronystagmography
ESRRB	Estrogen-Related Receptor Beta
FAM136A	Family With Sequence Similarity 136 Member A
FANC	Fanconi anemia complementation group
FAT4	FAT atypical cadherin 4
FMD	Familial Ménière’s disease
GJB2	Gap Junction Protein Beta 2
GPX1	Glutathione peroxidase-1
GUSB	Glucuronidase beta
GWAS	Genome-wide association studies
HCFC1	Host Cell Factor C1
HLA	Human Leukocyte Antigen
HMX2	H6 Family Homeobox 2
HRH4	Histamine Receptor H4
HSPs	Heat shock proteins
HSPA1A	Heat Shock Protein Family A Member 1A
IFN-γ	Interferon-gamma
IL1A	Interleukin-1 alpha
IL1B	Interleukin-1 beta
KCNE	Potassium Voltage-Gated Channel Subfamily E
KIRs	Killer immunoglobulin-like receptors
KNTCI	Kinetochore associated 1
LPIN2	Lipin-2
MD	Ménière’s disease
MEFV	Mediterranean FeVer
MHC	Major histocompatibility complex
MICA	MHC class I chain-related gene A
MIF	Macrophage Migration Inhibitory Factor
NBAS	Neuroblastoma Amplified Sequence
NFKB1	Nuclear Factor Kappa B Subunit 1
NK cells	Natural Killer cells
NOS	Nitric Oxide Synthase
NOX3	NADPH oxidase 3
NTN4	Netrin-4
OVCH1	Ovochymase 1
OTOG	Otogelin
OTOP1	Otopetrin 1
PARP-1	Poly(ADP-ribose) polymerase 1
PON	Paraoxonase 1
PTPN22	Protein Tyrosine Phosphatase Non-Receptor Type 22
PRKCB	Protein Kinase C Beta
RANTES	Regulated upon Activation, Normal T cell Expressed and Secreted
RAB27A	Member RAS Oncogene Family
RAG2	Recombination Activating Gene 2
ROS	Reactive oxygen species
RNF31	RING finger protein 31
SEMA3D	Semaphorin 3D
SIK1	Salt-Inducible Kinase 1
SLC6A7	Solute carrier family 6 member 7
SLC26A4	Solute carrier family 26 member 4
SLC26A5	Solute carrier family 26 member 5
SLC8A1	Solute Carrier Family 8 Member A1
SMD	Sporadic Ménière’s disease
SNHL	Sensorineural hearing loss
SNP	Single-nucleotide polymorphism
SOD	Superoxide dismutase
TLR3	Toll-like receptor 3
TLR10	Toll-like receptor 10
TMEM55B	Transmembrane Protein 55B
TMIE	Transmembrane Inner Ear
TNF-α	Tumor necrosis factor
USH1G	Usher syndrome type-1G protein
VUS	Variants of uncertain significance
WES	Whole exome sequencing
WGS	Whole Genome Sequencing
ZNF91	Zinc Finger Protein 91
